# Natural products from medicinal plants in Asia and the Pacific for RNA viruses: Hercules’ fifth labour

**DOI:** 10.1080/13880209.2022.2130944

**Published:** 2022-10-28

**Authors:** Mazdida Sulaiman, Vladimir Zarubaev, Chandramathi Samudi, Mohammed Rahmatullah, Khoshnur Jannat, Alok Paul, Mogana Rajagopal, Malarvili Salvaraj, Veeranoot Nissapatorn, Monica Suleiman, Mark Butler, Christophe Wiart

**Affiliations:** a Department of Chemistry, Faculty of Science, University of Malaya, Kuala Lumpur, Malaysia; b Department of Virology, Pasteur Institute of Epidemiology and Microbiology, Saint Petersburg, Russian Federation; c Department of Medical Microbiology, University of Malaya, Kuala Lumpur, Malaysia; d Department of Biotechnology and Genetic Engineering, University of Development, Dhaka, Bangladesh; e School of Pharmacy and Pharmacology, University of Tasmania, Hobart, Australia; f Faculty of Pharmaceutical Sciences, UCSI University, Kuala Lumpur, Malaysia; g School of Allied Health Sciences, World Union for Herbal Drug Discovery (WUHeDD) and Research Excellence Center for Innovation and Health Products (RECIHP), Walailak University, Nakhon Si Thammarat, Thailand; h Institute for Tropical Biology and Conservation, University Malaysia Sabah, Kota Kinabalu, Malaysia; i MSBChem Consulting, Brisbane, Australia

## Abstract

**Context:**

The emergence of zoonotic viruses in the last decades culminating with COVID-19 and challenges posed by the resistance of RNA viruses to antiviral drugs requires the development of new antiviral drugs.

**Objective:**

This review identifies natural products isolated from Asian and Pacific medicinal plants with i*n vitro* and *in vivo* antiviral activity towards RNA viruses and analyses their distribution, molecular weights, solubility and modes of action.

**Materials and methods:**

All data in this review was compiled from Google Scholar, PubMed, Science Direct, Web of Science, ChemSpider, PubChem and library search from 1961 to 2022.

**Results:**

Out of about 350 molecules identified, 43 phenolics, 31 alkaloids, and 28 terpenes were very strongly active against at least one type of RNA virus. These natural products are mainly planar and amphiphilic, with a molecular mass between 200 and 400 g/mol and target viral genome replication. Hydroxytyrosol, silvestrol, lycorine, tylophorine and 12-*O*-tetradecanoylphorbol 13-acetate with IC_50_ below 0.01 µg/mL and selectivity index (S.I.) above 100 have the potential to be used for the development of anti-RNA virus leads.

**Discussion and conclusions:**

The medicinal plants of Asia and the Pacific are a rich source of natural products with the potential to be developed as lead for the treatment of RNA viral infections.

**ARTICLE HISTORY**
Received 23 May 2022
Revised 21 September 2022
Accepted 23 September 2022

## Introduction

Waves of zoonotic RNA virus pandemics have been infecting humans for about the last 100 years starting with the Spanish flu of 1918, followed more recently by the Middle East respiratory syndrome coronavirus (MERS-CoV; with a mortality rate of 35.4%) and since December 2019, COVID-19 (coronavirus disease 2019) caused by the severe acute respiratory virus-associated coronavirus 2 (SARS-CoV-2) (Ye et al. 2020). Although vaccines afford good protection against these viruses, populations are left without effective chemotherapeutic defence until vaccines are developed; as a result, there is a dramatic need to find antiviral lead compounds to develop into innovative antiviral drugs. Such a wonder molecule could come from nature, and in particular, the flowering plants of Asia and the Pacific, which are the oldest, largest and richest on Earth.

Flowering plants also called Angiosperms are organised into 11 major taxa or Clades distributed into three groups: (i) Basal Angiosperms including Protomagnoliids, Magnoliids, Monocots and Eudicots; (ii) Core Angiosperms covering the Core Eudicots, Rosids, Fabids and Malvids, and the Upper Angiosperms that include the Asterids, Lamiids and Campanulids (Haston et al. 2009). Within each Clades, plants yield specific types of secondary metabolites to control phytopathogenic microbes including viruses. These antimicrobial natural products fall into two main groups: phytoanticipins and phytoalexins. Phytoanticipins are antimicrobial compounds that are present before the challenge by phytopathogenic microbes, such as tannins or inactive immediate precursors stored in healthy tissues as flavonoid glycosides which get converted into antimicrobial metabolites known as phytoalexins.

The discovery of antivirals from plant phytoalexins requires an understanding of viral replication which includes six stages (Samji 2009). These are: (i) attachment of virion to host cell, (ii) fusion and entry of virion with the cytoplasmic membrane of the host cell, (iii) uncoating of the virus and release of the viral contents in the host cell, (iv) building of new viral genome and

CONTACT Christophe Wiart christophewiart@ums.edu Institute of Tropical Biology and Conservation, University Malaysia Sabah, 88400 Kota Kinabalu, Malaysia macromolecules using the host cell, (v) assembly of viral genome and macromolecules into viral progeny, and (vi) release of viral progeny that can often induce host cell death. For RNA viruses to produce viral progeny, the viral genome must be translated, i.e., converted into proteins that will further associate with viral particles. Single-stranded (+)-RNA genome of single-stranded (+)-RNA viruses are translated directly by host cell ribosomes into a large protein (or polyprotein) that is cleaved by viral proteases into structural proteins and enzymes that catalyses the synthesis of a complementary strand of virus-specific RNA. If the single-stranded (+)-RNA viruses are equipped with reverse transcriptase, such as the Human Immunodeficiency virus (HIV), a complementary single-stranded DNA is produced which is further converted into double-stranded DNA that integrates into the host DNA permanently. Human pathogenic (+) RNA viruses are found in the family Coronavidae, such as SARS-CoV-2, Flaviviridae, such as the Dengue virus (DV) and Picornaviriae, such as the Human Rhinovirus. Single-stranded (−)-RNA genome is replicated by the host cell and the viral RNA-dependent RNA polymerase to produce single-stranded (+)-RNA that serves as a messenger RNA as well as intermediate for further synthesis of virus-specific genome (−)-RNA. Likewise, for double-stranded RNA viruses, RNA-dependent RNA polymerase uses the negative strand of the double-stranded RNA to generate a single-stranded (+)-RNA. Human pathogenic (−)-RNA viruses are found in the family Paramyxoviridae and Orthomyxoviridae, such as the influenza virus.

The search for natural products that inhibit the replication of RNA viruses also require the measurement of the antiviral strength *in vitro* that is quantitively based on the $IC_{50}$ and selectivity index (S.I.). Ideal candidates have low $IC_{50}$ and large S.I. Consider that natural products from plants with antiviral activity have often some levels of cytotoxicity. Hence, a good antiviral property can be observed *in vitro* and at the same time toxicity towards cultured host cells. The generally accepted threshold of S.I. in the area of antiviral development is 10, i.e., if a compound demonstrates S.I. of 10 or higher it is considered prospective (Smee et al. 2017).

This review addresses the following points in regard to the natural products with anti-RNA virus activity identified from the flowering plants of Asia and the Pacific: distribution, strength, spectrum of activity, influence of molecular mass and solubility, mechanisms of action, structure-activity, selectivity index, *in vivo* activity and clinical potentials. For about the last 80 years, an enormous body of experimental evidence has been generated with the hope to find anti-RNA virus natural products of clinical value in plants globally. This review provides a taxonomical, phytochemical, biomolecular and physicochemical rationale to facilitate the discovery of leads for the treatment of RNA virus infections.

## (+)-RNA viruses

### Rotavirus (RTV)

RTV is responsible for life-threatening gastroenteritis in young unvaccinated children. The virion VP4 protein binds *via* to the sialo-glucan receptor or histo-blood antigens of the host cell and the viral DNA is translated into (+)-RNA (Desselberger 2017). From the medicinal plants of Asia and the Pacific, phenolics moderately inhibit RTV *in vitro* such as theaflavin-3′-gallate (**97**) from *Camellia sinensis* (L.) Kuntze (Theaceae) ($IC_{50}$ 5.5 µg/mL) (Clark et al. 1998), multibiphenyl B (Gao et al. 2016), carvacrol (Pilau et al. 2011) and vitexin (Knipping et al. 2012).

### Feline calicivirus (FCV)

The hydroxycinnamic acid derivative ferulic acid at 500 µg/mL nullified FCV-F9 titres (Joshi et al. 2015).

### Enterovirus 71 (EV-71)

EV-71 accounts for the hand, foot, and mouth disease that has the potential to develop into life-threatening neurological disorders in young children. The virion surface VP1 protein binds to the scavenger receptor B2 and human P-selectin glycoprotein ligand-1 of host cells and the viral (+)-RNA is used for the synthesis of a polyprotein (Kuo and Shih 2013). A number of natural products are very strong suppressors of this virus (Table 1).

### Phenolics

Phenolics with the strongest activity against EV-71 are gallic acid (**1**) ($IC_{50}$ 0.7 µg/mL; S.I. 99.5) (Choi et al. 2010) from *Lagerstroemia speciosa* (L.) Pers. (Lythraceae), baicalein ($IC_{50}$ 2.2 µg/mL; S.I 20) (Lalani et al. 2020), the flavones chrysosplenetin (**14**) and penduletin (**15**) in *Laggera pterodonta* (DC.) Sch. Bip. ex Oliv. (Asteraceae) with the $IC_{50}$ values of 0.2 and 0.3 µM and the S.I. of 69.5 and 200.5, respectively (Zhu et al. 2011). Aloe-emodin (**2**) from *Aloe vera* (L.) Burm. f. (Asphodelaceae) restrained EV-71 with the $IC_{50}$ value of 0.1 µg/mL and a S.I. of 18800 (Lin et al. 2008). Other phenolics active against EV-71 are oblongifolin J (Zhang et al. 2014), chlorogenic acid (Lin et al. 2008), norwogonin, oroxylin A, mosloflavone (Choi et al. 2016), chrysin, formononetin, euxanthone (Zhang et al. 2014), geraniin (**43**) and corilagin (Yeo et al. 2015). Note that gallic acid can be extracted using phosphoric and aminic extractants (Joshi et al. 2021).

### Alkaloids

Lycorine (**3**) suppressed EV-71 with an $IC_{50}$ of 0.4 µg/mL ($CC_{50}$ 48.5 µg/mL) and given intraperitoneally at a dose of 1 mg/kg twice for 7 d to mice experimentally infected with EV-71 prolonged the survival time by 45%. Other alkaloids that inhibit EV-71 are berberine (**98**), harmine, camptothecin, and matrine.

### Terpenes

The ursane triterpene ursolic acid (**4**) from *Ocimum basilicum* L. (Lamiaceae) inhibited EV-71 with the $EC_{50}$ value of 0.5 µg/mL and a S.I. of 201 (Chiang et al. 2005). Other terpenes suppressing EV-71 are linalool (Chiang et al. 2005) and the norbisabolane sesquiterpene glycoside glochicoccinoside D from *Phyllanthus emblica* L. (Phyllanthaceae) ($IC_{50}$ 2.6 µg/mL). As for saponins, ginsenoside Rg2 from *Panax ginseng* C.A Meyer (Araliaceae) is strongly active (Song et al. 2014).

### Poliovirus (PV)

PV is responsible for neuromuscular atrophy in unvaccinated individuals. Lignans and anthraquinones have very strong

**Table 1.** Very strong inhibitors of RNA viruses from the medicinal plants of Asia and the Pacific.

| Virus | Family | Principle | S.I > 10 | LM | MM | HM | HP | AP | LP | *In vivo* |
|---|---|---|---|---|---|---|---|---|---|---|
| Non-enveloped, segmented, linear and double-stranded (+) RNA | | | | | | | | | | |
| RTV | Reoviridae | None | | | | | | | | |
| Non-enveloped, monopartite and linear single-stranded (+) RNA | | | | | | | | | | |
| FCV | Caliciviridae | None | | | | | | | | |
| EV-71 | Picornaviridae | Gallic acid (**1**) | | 170.1 | | | −0.8 | | | |
| | | Aloe-emodin (**2**) | ● | | 270.2 | | | 1.2 | | |
| | | Lycorine (**3**) | ● | | 287.3 | | | 3.1 | | |
| | | Ursolic acid (**4**) | ● | | | 456.7 | | | 5.8 | |
| PV | Picornaviridae | Euparin (**5**) | | | 216.1 | | | 3.6 | | |
| | | Chrysophanic acid (**6**) | | | 254.2 | | | 3.9 | | |
| | | Emodin (**7**) | | | 270.2 | | | 3.7 | | |
| | | Lycorine (**3**) | ● | | | 456.7 | | | 5.8 | |
| HRV | Picornaviridae | Silvestrol (**8**) | ● | | | 654.6 | | 2.1 | | |
| | | Axillarin (**9**) | | | 346.2 | | 0.6 | | | |
| | | Orobol 7-*O*-D-glucoside (**10**) | | | | 448.4 | | | | |
| | | Rhinacanthin E (**11**) | | | | 442.4 | | | | |
| | | Rhinacanthin F (**12**) | | | | 444.2 | | | | |
| | | Rhinacanthin G (**13**) | | | | 426.5 | | | | |
| CV | Picornaviridae | Chrysosplenetin (**14**) | | | 374.3 | | | 2.4 | | |
| | | Penduletin (**15**) | | | 344.3 | | | 2.6 | | |
| | | Rhinacanthin E (**11**) | | | | | | | | |
| | | Ursolic acid (**4**) | | | | 456.7 | | | 5.8 | |
| EMCV | Picornaviridae | None | | | | | | | | |
| Enveloped, monopartite, linear and single-stranded (+) RNA | | | | | | | | | | |
| PRRSV | Arteriviridae | None | | | | | | | | |
| HcoV-229E | Coronaviridae | Silvestrol (**8**) | ● | | | 654.6 | | 2.1 | | |
| | | Kuwanon G (**16**) | ● | | | 692.7 | | | 5.1 | |
| HcoV-NL63 | Coronaviridae | Tryptanthrin (**17**) | ● | 248.2 | | | | 2.4 | | |
| PEDV | Coronaviridae | Primulagenin A (**18**) | | | | 458.3 | | | 7.2 | |
| | | Schimperinone (**19**) | | | | 456.7 | | | 7.1 | |
| TGEV | Coronaviridae | Tylophorine (**20**) | ● | | | 393.4 | | 3.9 | | |
| HcoV-O43 | Coronaviridae | Fangchinoline (**21**) | | | | 608.7 | | 3.4 | | |
| SARS-CoV | Coronaviridae | Lycorine (**3**) | ● | | 287.3 | | | 3.1 | | |
| SARS-CoV2 | Coronaviridae | Panduratin A (**22**) | ● | | | 406.5 | | | 6.6 | |
| MHV | Coronaviridae | None | | | | | | | | |
| MERS | Coronaviridae | Silvestrol (**8**) | ● | | | 654.6 | 2.1 | | | |
| IBV | Coronaviridae | None | | | | | | | | |
| DV | Flaviridae | Pseudolycorine (**23**) | | | | 289.3 | | 0.3 | | |
| | | Lycoricidine (**24**) | | | | 291.2 | | −0.1 | | |
| | | Pancratistatin (**25**) | | | 325.2 | | −0.4 | | | |
| JEV | Flaviridae | Narciclasine (**26**) | | | 307.2 | | 0.09 | | | |
| | | Lycoricidine (**24**) | | | 291.2 | | −0.1 | | | |
| | | Pseudolycorine (**23**) | | | 289.3 | | 0.3 | | | |
| | | Pretazettine (**27**) | | | 331.3 | | 0.2 | | | |
| | | Pancratistatin (**25**) | | | 325.2 | | −0.4 | | | ● |
| YFV | Flaviridae | Naringenin (**28**) | | | 272.2 | | | 2.2 | | |
| | | Hesperitin (**29**) | | | 302.2 | | | 2.2 | | |
| | | Narciclasine (**26**) | | | 307.2 | | 0.09 | | | |
| | | Lycoricidine (**24**) | | | 291.2 | | −0.1 | | | |
| | | Pancratistatin (**25**) | | | 325.2 | | −0.4 | | | |
| WNV | Flaviridae | Lycorine (**3**) | | | 287.3 | | | 3.1 | | |
| ZIKV | Flaviridae | Silvestrol (**8**) | | | 654.6 | | | 2.1 | | |
| | | Diphyllin (**30**) | | | 380.3 | | | 2.5 | | |
| HCV | Flaviridae | Trachelogenin (**31**) | | | 388.4 | | | 1.4 | | |

(*continued*)

Table 1. Continued.

| Virus | Family | Principle | S.I > 10 | LM | MM | HM | HP | AP | LP | *In vivo* |
|---|---|---|---|---|---|---|---|---|---|---|
| | | Plumbagin (**32**) | ● | | 188.1 | | | 1.4 | | |
| | | Platycodin D (**33**) | | | | 1225.3 | −1.5 | | | |
| CHIKV | Togaviridae | Silvestrol (**8**) | | | | 654.6 | | 2.1 | | |
| | | 12-*O*-Tetradecanoyl Phorbol-13-acetate (**34**) | ● | | | 616.8 | | | 8.0 | |
| SINV | Togaviridae | None | | | | | | | | |
| MAYV | Togaviridae | Epicatechin (**35**) | | | 290.2 | | 0.5 | | | |
| SFV | Togaviridae | None | | | | | | | | |
| Enveloped, monopartite, linear, dimeric and single-stranded (+) RNA | | | | | | | | | | |
| HIV | Retroviridae | Hydroxytyrosol (**36**) | ● | 154.1 | | | 0.3 | | | |
| | | Justiprocumin B (**37**) | | | | | 731.2 | | | |
| | | Isopimpinellin (**38**) | | | 246.2 | | | 2.1 | | |
| | | *O*-Methylcedrelopsin (**39**) | | 274.3 | | | | | | |
| | | 4,5-di-*O*-Caffeoylquinic acid (**40**) | | | | 516.4 | −2.5 | | | |
| | | Calanolide A (**41**) | | | 370.4 | | | 4.7 | | |
| | | Hinokinin (**42**) | | | 354.3 | | | 3.1 | | |
| | | Geraniin (**43**) | | | | 952.6 | −0.06 | | | |
| | | Coclaurine (**44**) | ● | | 285.3 | | 0.2 | | | |
| | | Liensinine (**45**) | | | | 610.7 | | 3.8 | | |
| | | Neferine (**46**) | | | | 624.8 | | | | |
| | | Isoliensinine (**47**) | | | | 610.7 | | | | |
| | | Nornuciferine (**48**) | ● | | 281.3 | | | 2.7 | | |
| | | Drymaritine (**49**) | ● | | 250.2 | | | | | |
| | | Wilfortrine (**50**) | | | | 873.8 | | 3.6 | | |
| | | Wilfordin (**51**) | | | | 883.8 | | 4.4 | | |
| | | Hypoglaunine A (**52**) | | | | 873.8 | | 3.6 | | |
| | | Hypoglaunine B (**53**) | | | | 857.2 | | 4.6 | | |
| | | 6-Acetonyldihydronitidine (**54**) | | | | 404.4 | | | | |
| | | Decarine (**55**) | | | 319.3 | | | 3.2 | | |
| | | Dihydroavicine (**56**) | | | 333.3 | | | 4.3 | | |
| | | Haplopine (**57**) | | | 245.2 | | 0.4 | | | |
| | | 4-Methoxy-1-methyl −2-Quinolone (**58**) | | | 175.1 | | | 2.7 | | |
| | | *O*-Methyltembamide (**59**) | | | 285.3 | | | | | |
| | | Oxyavicine (**60**) | | | 347.3 | | | | | |
| | | Tembamide (**61**) | | | 271.3 | | | 2.1 | | |
| | | 16β-17-Dihydroxy *ent*-Kauran-19-oic acid (**62**) | | | 336.4 | | 0.8 | | | |
| | | Triptolide (**63**) | | | 360.1 | | 0.6 | | | |
| | | Excoecafolin B (**64**) | ● | | | 586.8 | | | | |
| | | Excoecafolin C (**65**) | ● | | | 570.8 | | | | |

| Virus | Family | Principle | S.I. > 10 | LM | MM | HM | HP | AP | LP |
|---|---|---|---|---|---|---|---|---|---|
| | | 12-Deoxyphorbol 13-(3*E*,5*E*) Decadienoate (**66**) | | | | 498.6 | | | |
| | | Stelleralide A (**67**) | | | | 712.8 | | | 6.9 |
| | | Stelleracin B (**68**) | | | | | | | |
| | | Stelleracin C (**69**) | | | | | | | |
| | | Trigonothyrin F (**70**) | ● | | | | | | |
| | | Moronic acid (**71**) | | | | 454.6 | | 5.5 | |
| | | Limonin (**72**) | | | | 470.5 | | 1.6 | |
| | | Actein (**73**) | ● | | | 676.8 | | 4.4 | |
| | | Oleandrin (**74**) | | | | 576.7 | | 2.4 | |
| | | Biyouyanagin A (**75**) | ● | | | 474.6 | | | 7.4 |

(*continued*)

**Table 1.** Continued.

| Virus | Family | Principle | S.I. > 10 | LM | MM | HM | HP | AP | LP |
|---|---|---|---|---|---|---|---|---|---|
| | | Clausenolide-1-ethyl ether (**76**) | | | | 488.6 | | | |
| Enveloped, monopartite, linear and single-stranded (−) RNA | | | | | | | | | |
| EBOV | Filoviridae | Silvestrol (**8**) | | | | 654. | | | |
| | | Oleandrin (**74**) | | | | 526.7 | | 2.4 | |
| NDV | Paramyxoviridae | None | | | | | | | |
| MEV | Paramyxoviridae | None | | | | | | | |
| HPIV-3 | Paramyxoviridae | Caesalmin B (**77**) | | | 388.5 | | | | |
| | | Bonducellpin D (**78**) | | | | 404.5 | | | |
| | | 1α,5α,6α,7β,14β-Pentahydroxyvouacapane | | | | | | | |
| | | 1α,5α-Dihydroxy-14β-methoxy-6α,7β-diacetoxyvouacapane (**79**) | | | | | | | |
| | | Friedelin (**80**) | | | | 426.7 | | | 10 |
| RSV | Pneumoviridae | Broussonin A (**81**) | | | 258.3 | | | | |
| | | 3,5-di-*O*-Caffeoylquinic acid (**82**) | | | | 516.4 | | | |
| | | Nyasol (**83**) | | | 252.3 | | | | |
| | | 4′-*O*-Methylnyasol (**84**) | | | 266.3 | | | | |
| | | Dammarenolic acid (**85**) | ● | | 458.7 | | | | |
| | | Dysoxylin D (**86**) | | | | | | | |
| VSV | Rhabdoviridae | None | | | | | | | |
| Enveloped, segmented, linear and single-stranded (−) RNA | | | | | | | | | |
| LASV | Arenaviridae | None | | | | | | | |
| PTV | Bunyaviridae | Narciclasine (**26**) | | | 307.2 | | 0.09 | | |
| | | Lycoricidine (**24**) | | | 291.2 | | −0.1 | | |
| | | Pseudolycorine (**23**) | | | 289.3 | | 0.3 | | |
| | | Pretazettine (**27**) | | | 331.3 | | 0.2 | | |
| RFV | Phenuiviridae | Pseudolycorine (**23**) | | | 289.3 | | 0.3 | | |
| | | Pretazettine (**27**) | | | 331.3 | | 0.2 | | |
| | | Lycoricidine (**24**) | | | 291.2 | | −0.1 | | |
| | | Pancratistatin (25) | | | 325.2 | | −0.4 | | |
| IV | Orthomyxoviridae | *epi*-Conferdione (**87**) | | | 394.5 | | | | |
| | | 10′*R*-Acetoxy-11′-hydroxyumbelliprenin (**88**) | | | | | | | |
| | | Methyl galbanate (**89**) | | | | 412.5 | | | 6.3 |
| | | Farnesiferol C (**90**) | | | 382.4 | | | | 5.1 |
| | | Quercetin (**91**) | | | | | | | |
| | | Rutin (**92**) | | | | 610.5 | −1.7 | | |
| | | Baicalin (**93**) | ● | | | 444.3 | −4.5 | | |
| | | Sappanone A (**94**) | | | 284.2 | | | | |
| | | Embelin (**95**) | | | 294.3 | | 0.5 | | |
| | | Hypericin (**96**) | | | | 504.4 | | | 7.1 |
| | | 1,3,4,6-Tetra-*O*-galloyl-β-D-glucopyranoside (**97**) | | | | 788.5 | | | |
| | | Theaflavin-3,3′-digallate (**98**) | | | 868.7 | | 0.3 | | |
| | | Berberine (**99**) | | | | 336.3 | | −1.3 | |
| | | (-)-Thalimonine (**100**) | ● | | | | | | |
| | | Hirsutine (**101**) | ● | | 368.5 | | | 2.6 | |
| | | Reynoudiol (**102**) | ● | | | | | | |

Molecular mass (g/mol), LM: low molecular mass; MM: medium molecular mass; HM: high molecular mass
LogD at pH 7.4; HP: hydrophilic; AP: amphiphilic; LP: lipophilic activities against PV (Table 1). The benzofuran lignan euparin (**5**) from *Eupatorium chinense* L. (Asteraceae) inhibited PV-1, 2 and 3 with the $EC_{50}$ values of 0.4, 0.1 and 0.1 µg/mL, respectively and the S.I. of 284.9, 1068 and 854.7, respectively. The anthraquinone chrysophanic acid (**6**) from *Rheum australe* D. Don (Polygonaceae) suppressed PV-3 with the $EC_{50}$ value of 0.02 µg/mL. Aloe-emodin (**2**) and emodin (**7**) repressed PV-3 with the $IC_{50}$ values of 0.5 and 0.2 µg/mL. Other phenolics with anti-PV activities are methyl-5-hydroxydinaphthol[1,2–2″,3″]furan-7,12-dione-6-carboxylate, 5,6,7-trimethoxyflavone, ermanin and calycopterin. Lycorine (**3**) very strongly inhibited PV-1 with an $IC_{50}$ value of 0.06 µg/mL and a S.I. of 24. Other alkaloids that repress PV are isoboldine, heliotrine, subulacine-*N*-oxide (Singh et al. 2002) and atropine (Yamazaki and Tagaya 1980).

## Human rhinovirus (HRV)

Individuals infected with HRV develop common cold. The virion binds to ICAM-1 and LDL-receptors at the surface of the nasal epithelial cells and after internalisation and disintegration of capsid, (+)-RNA is translated into a polyprotein that is cleaved into structural proteins and non-structural proteins. A number of natural products are very strong suppressors of this virus (Table 1), such as the flavonol axillarin (**9**) ($EC_{50}$ 0.6 µg/mL) from *Inula japonica* Thunb. (Asteraceae) and the isoflavone glycoside orobol 7-*O*-D-glucoside (**10**) ($IC_{50}$ 0.5 µg/mL; S.I > 555.5) in *L. speciosa* (Choi et al. 2010). Other examples are the dibenzylbutane lignans rhinacanthins E (**11**) and F (**12**), and the naphthoquinone rhinacanthin G (**13**) ($IC_{50}$ 0.2, 0.2 and 0.9 µg/mL, respectively) (Ngoc et al. 2019), silvestrol (**8**) in the genus *Aglaia* Lour. ($IC_{50}$ 100 nM; S.I. >100) (Müller et al. 2018), as well as kaempferol-3-*O*-[2″,6″-di-*O*-*E*-*p*-coumaroyl]-β-D-glucopyranoside ($IC_{50}$ 1.2 µg/mL) (Kim et al. 2019). The cycloartane triterpene castaartancrenoic acid E strongly active ($IC_{50}$ 5.5 µg/mL). Gallic acid (**1**) (Choi et al. 2010), paeonol and ellagic acid repress HRV.

## Coxsackievirus (CV)

CV accounts for myocarditis. The virion binds to the decay accelerating factor (DAF/CD55) and viral (+)-RNA is translated into a polyprotein further cleaved into structural and non-structural proteins by viral proteases 2 $A^{pro}$ and 3 $C^{pro}$. No treatment is available. A number of natural products are very strong suppressors of this virus (Table 1).

## Phenolics

The phenolic glycoside salidroside from *Rhodiola rosea* L. (Crassulaceae) given daily at 80 mg/kg/d for 7 d orally to mice infected with CVB3 prevented cardiac insults (Wang et al. 2009). Other examples include 2-*O*-caffeoyl-(+)-allohydroxycitric (Corthout et al. 1992) and bergapten. Examples of flavonols strongly active against CV are chrysosplenetin (**14**) and penduletin (**15**) ($IC_{50}$ 0.2 and 0.3 µM, respectively) (Zhu et al. 2011), chrysin ($EC_{50}$ 2 µM) (Song et al. 2015) and oroxylin ($IC_{50}$ 3.1 µg/mL) from *Oroxylum indicum* L. (Bignoniaceae) (Kwon et al. 2016). Other instances of flavonoids that repress CV are taxifolin (Bakay et al. 1968), ermanin (Chiang et al. 2005), galangin (Meyer et al. 1997), 5,4′-dihydroxy-7,3′-dimethoxyflavone ($IC_{50}$ 1.8 µM) (Ban et al. 2018), myricitrin, desmanthin (Van Nguyen et al. 2014), calycosin and calycosin-7-*O*-β-D-glucopyranoside (Zhu et al. 2009). Dihydroquercetin (taxifolin) administered intraperitoneally at a dose of 150 mg/kg/d for 5 d reduced CVB4 virus titre in mouse pancreas (Galochkina et al. 2016) whereas calycosin-7-*O*-β-D-glucopyranoside at 24 mg/kg/d for 14 d increased the survival rate of mice infected with CV (Nancy Strain) (Zhu et al. 2009).

Lignans are active *in vivo*, such as manassantin B from *Saururus chinensis* (Lour.) Baill. (Saururaceae) given intraperitoneally to mice at the dose of 2.5 mg/kg (Song et al. 2019). Rhinacanthin E (**11**) from *Rhinacanthus nasutus* (L.) Kurz. (Acanthaceae) yielded an $IC_{50}$ value of 0.03 µM and S.I. >1000 (Ngoc et al. 2019). In this plant, rhinacanthin G (**13**) is a strong inhibitor (Ngoc et al. 2019).

Emodin (**7**) given orally to mice at a dose of 30 mg/kg/d for 14 d evoked levels of protection against CV (Liu et al. 2013). As for tannins, they are in general moderate CV suppressors *in vitro* such as epicatechin-(2β→*O*→7,4β→6)-epicatechin-(2β→*O*→7, 4β→6)- (Xue et al. 2010), corilagin (Yeo et al. 2015) and geraniin (**43**) (Corthout et al. 1992).

## Alkaloids

7-Angeloyl heliotrine (Singh et al. 2002), 4,5-dimethoxycanthin-6-one (Apers et al. 2002), and berberine (**98**) (Zeng et al. 2020) are moderate CV inhibitors.

## Terpenes

In general, terpenes are moderately active against CV, such as linalool (Chiang et al. 2005), 7α-hydroxycallitrisic acid (Zhang, Liu, et al. 2013; Zhang, Li, et al. 2013), jiadifenoic acid M (Zhang et al. 2014), mansonone E and H (Zhang, Liu, et al. 2013; Zhang, Li, et al. 2013), phyllaemblicin B and simalikalactone (Apers et al. 2002). Of interest, ursolic acid (**4**) yielded the $EC_{50}$ value 0.4 µg/mL and the S.I. of 251.3 (Chiang et al. 2005). Cornaceae plants yield iridoid glycosides, such as morroniside (*Cornus officinalis* Sieb. and Zucc.) active *in vivo* (20 mg/kg intraperitoneally) (Li et al. 2019) and the cadinane sesquiterpene 2,7-dihydroxycadalene (*Alangium chinense* (Lour.) Harms) ($IC_{50}$ 1.4 µM) (Zhang, Liu, et al. 2013; Zhang, Li, et al. 2013).

## Encephalomyocarditis virus (EMCV)

EMCV is zoonotic from rodents and accounts for fatal neurological and cardiac insults in apes, pigs, and human (Oberste et al. 2009). No treatment exists for this virus. The virion follows a classic cell cycle including cleavage of polyprotein by viral 3CProtease (3 $C^{Pro}$). The iridoid glycoside arbortristoside A in *Nyctanthes arbor-tristis* L. (Oleaceae) strongly suppressed EMCV.

## Porcine reproductive and respiratory syndrome virus (PRRSV)

PRRSV accounts for respiratory infections and sterility of unvaccinated pigs. The virion binds to the sialo-adhesin receptor of macrophages and release a single-stranded (+)-RNA translated further into a polyprotein that is cleaved into structural proteins and enzymes, the latter catalysing the synthesis of a double-stranded RNA (Wang, Dong, et al. 2021; Wang, Zeng, et al. 2021). Phenolics in *C. sinensis* repressed PRRSV at the concentration of 10 μg/mL (Wang, Dong, et al. 2021; Wang, Zeng, et al. 2021) and the kaurane diterpene methyl pothoscandensate is a weak inhibitor (Liu et al. 2012).

### The human coronavirus 229E (HCoV 229E)

Individuals infected with HCoV 229Edevelop common cold. The virion binds onto the human aminopeptidase N receptor of host cells *via* the spike proteins and viral RNA is released in the cytoplasm and translated into viral proteins (Yeager et al. 1992). A number of phenolics are very strong suppressors of this virus (Table 1), such as silvestrol (**8**) ($IC_{50}$ 3 nM) (Müller et al. 2018), the prenylated flavone kuwanon G (**16**) in *Morus alba* L. (Moraceae) ($EC_{50}$ 0.1 μg/mL; S.I. 86.7) (Thabti et al. 2020). Prenylated xanthones in the genus *Calophyllum* L. (Calophyllaceae) strongly restrain HCoV-229E, such as blancoxanthone and pyranojacareubin with the $IC_{50}$ values of 3 and 15 μg/mL, respectively (Shen et al. 2005). In the Euphorbiaceae, *Euphorbia neriifolia* L. yields 3β-friedelanol, 3β-acetoxy friedelane, friedelin (**80**) and epitaraxerol (Chang et al. 2012). Moderate to weak inhibitors are naringenin (**28**) (Clementi et al. 2021) and phillyrin (Ma et al. 2020).

### Human coronavirus NL63 (HCoV NL63)

HCoV NL63 accounts for conjunctivitis, fever and bronchiolitis. The virion spike proteins bind to angiotensin-converting enzyme (Chiu et al. 2005). Alkaloids repress HCoV-NL63 *in vitro* such as the quinazoline alkaloid tryptanthrin (**17**) from *Indigofera tinctoria* L. (Fabaceae) ($IC_{50}$ 0.06 μg/mL; S.I. >6600) (Tsai et al. 2020) and the steroidal alkaloid conessine (Lima et al. 2021). Phenolics are moderate to weak HCoV-NL63 repressors as seen with caffeic acid, gallic acid (**1**) and chlorogenic acid (Weng et al. 2019).

### Porcine epidemic diarrhoea virus (PEDV)

Pigs infected with the PEDV develop diarrhoea, vomiting, and fatal dehydration. The virion spike proteins bind to aminopeptidase N receptors of host cells to release a single-stranded (+)-RNA translated into a polyprotein (Lee 2015). The oleanane triterpenes primulagenin A (**18**), 3β-hydroxy-28-noroleana-12,17-dien-16-one, and schimperinone (**19**) in *Camellia japonica* L. (Theaceae) very strongly inhibited PEDV with the $IC_{50}$ values of 0.06, 0.2, and 0.06 μM, respectively, and S.I. of 12.9, 32.7 and 44.5, respectively (Yang, Dhodary, et al. 2015; Yang, Ha, et al. 2015). Example of strong inhibitors are quercetin-7-rhamnoside, divaricoumarin A (Yang, Dhodary, et al. 2015; Yang, Ha, et al. 2015) and jubanine H (Kang et al. 2015).

### Transmissible gastroenteritis virus (TGEV)

Another α-coronavirus infecting pigs is TGEV which is very strongly inhibited by the phenanthroindolizidine alkaloid tylophorine (**20**) in plants from the genus *Ficus* L. (Moraceae) with an $IC_{50}$ value as low as 58 nM and a S.I. above 1715 (Yang, Lee, et al. 2010; Yang, Li, et al. 2010).

### Human coronavirus O43 (HCoV O43)

HCoV-O43 originates from rodents. The virion binds to 9-*O*-acetylated sialic acid of host cells and undergoes similar cell cycle as α-coronaviruses (Ye et al. 2020). Inhibitors of HCoV-O43 are mainly alkaloids. Menispermaceae plants yield bisbenzylisoquinolines with strong potencies against HCoV-O43, such as tetrandrine ($IC_{50}$ 0.3 μM), fangchinoline (**21**) ($IC_{50}$ 1 μM) (Charlton et al. 2020) and cepharanthine from *Stephania rotunda* Lour. ($IC_{50}$ 0.8 μM; S.I. 13.6) (Kim et al. 2019). Examples of strong inhibitors are conessine from *Holarrhena pubescens* Wall. (Apocynaceae) ex G. Don ($EC_{50}$ 2.3 μM) (Lima et al. 2021) and kuranone from *Gentiana macrophylla* Pallas (Gentianaceae) ($IC_{50}$ 3.4 μM) (Min et al. 2020).

### Severe acute respiratory syndrome-associated coronavirus (SARS-CoV)

SARS-CoV is responsible fever, cough and breathing difficulties and originated from the consumption of masked palm civets and racoon dogs in China (Ye et al. 2020).

#### Coumarins

*Boenninghausenia albiflora* (Hook.) Rchb. ex Meisn. (Rutaceae) yields leptodactylone and the furanocoumarin rutamarin (Yang et al. 2007) that are moderately active against SARS-CoV. Note that plants in the genus *Heracleum* L. (Apiaceae) are a rich source of furanocoumarins (Hosseinzadeh et al. 2019).

#### Flavonoids

Baicalein in *Scutellaria baicalensis* Georgi (Lamiaceae) (Su et al. 2020) is a SARS-CoV repressor as well as luteolin (Yi et al. 2004), hesperetin (Lin et al. 2005), herbacetin (Jo et al. 2020), tomentin E (Cho et al. 2013), amentoflavone (Ryu et al. 2010), bavachinin and isobavachalcone (Kim et al. 2014).

#### Lignans

Dibenzylbutyrolactone lignans are moderate inhibitors of SARS-CoV such as hinokinin (**42**) and savinin (Wen et al. 2007).

#### Anthraquinones

Emodin (**7**) and rhein are moderate inhibitors of SARS-CoV (Ho et al. 2007).

#### Tannins

1,2,3,6-Tetra-*O*-galloyl-β-D-glucose from *Rhus javanica* L. (Anacardiaceae) strongly inhibited SARS-CoV with the $IC_{50}$ value 4.5 μg/mL (Yi et al. 2004). As for condensed tannins, procyanidin A2, procyanidin B1 and cinnamtannin B1 from *Cinnamomum cassia* (L.) J. Presl (Lauraceae) moderately suppressed SARS-CoV (Zhuan et al. 2009). In a subsequent study, proanthocyanidins from the follicles of *Zanthoxylum piperitum* (L.) DC. (Rutaceae) yielded the $IC_{50}$ of 5.5 μg/mL (Maeda et al. 2011). Other examples are theaflavin-3,3′-digallate (**97**) (Chen et al. 2005) and epigallocatechin gallate.

#### Alkaloids

The Amaryllidaceae alkaloid lycorine (**3**) from *Crinum asiaticum* L. (Amaryllidaceae) suppressed SARS-CoV with an $EC_{50}$ value as low as 15.7 nM and a S.I. of 954. Indolizidine alkaloids (Yang, Lee, et al. 2010; Yang, Li, et al. 2010), caffeine and theophylline are active (Chen et al. 2005).

### Terpenes

Iridoid glycosides, such as swertiamarin (Li et al. 2016) and cornuside are inhibitors of SARS-CoV (Zhang et al. 2021) as well as lamiaceous diterpenes, such as cryptotanshinone (*Salvia miltiorrhiza* Bunge) (Park et al. 2012), the ergostane withanone (Balkrishna et al. 2021) and glycyrrhizin.

### Miscellaneous

### Sinigrin

Severe acute respiratory syndrome-associated coronavirus – 2 (SARS-CoV-2)

SARS-CoV-2 may have originated from the consumption of pangolin or bats in China and is responsible for life-threatening pneumonia (Lin et al. 2005; Ye et al. 2020).

### Phenolics

Phenolics suppress SARS-CoV-2 *in vitro* and some of them very strongly, such as the prenylated chalcone panduratin A (**22**) from *Boesenbergia rotunda* (L.) Manfs. (Zingiberaceae) ($IC_{50}$ 0.8 µM) (Kanjanasirirat et al. 2020). Strong inhibitors are rhodomyrtone from *Rhodomyrtus tomentosa* (Aiton) Hassk (Myrtaceae) ($EC_{50}$ 0.4 µM) (Tang, Zhang, et al. 2020). The lignan phillyrin in the genus *Forsythia* Vahl (Oleaceae) is moderately active (Ma et al. 2020).

### Terpenes

*Artemisia vulgaris* L. (Asteraceae) yields the sesquiterpene artemisinin that given to COVID-19 patients at 125 mg with piperaquine 750 mg on the first day followed artemisinin 62.5 mg and piperaquine 375 mg/d for 6 d evoked clearance of the virus and levels of pulmonary protection (Li et al. 2021). *Andrographis paniculata* (Burm. f.) Wall. ex Nees (Acanthaceae) produces the labdane diterpene andrographolide which when given orally to COVID-19 patients at the dose of 20 mg 3 times per day for 5 d prevented the development of pneumonia (Wanaratna et al. 2021). The cardiac glycoside oleandrin (**74**) from *Nerium oleander* L. (Apocynaceae) yielded an $EC_{50}$ value of 11.9 ng/mL (Plante et al. 2021). Oleandrin (**74**) given to hamsters (sub-lingually at the dose of 25 µL of a 130 µg/mL solution once daily for 5 d) evoked some levels of protection (Plante et al. 2021).

### Mouse hepatitis virus (MHV)

Quercetin and conessine are moderate inhibitors of MHV (Chiow et al. 2016; Shen et al. 2019).

### Middle East respiratory syndrome coronavirus (MERS-CoV)

MERS-CoV is a zoonotic virus that produces cough, fever, myalgia, pneumonia and respiratory distress (Ye et al. 2020). The virion anchors to host cell by the dipeptidyl peptidase surface enzyme (Ye et al. 2020). Silvestrol (**8**) restrained MERS-CoV with an $IC_{50}$ value as low as 1.3 nM and a S.I. above 100 (Müller et al. 2018). Flavonoids moderately inhibit MERS-CoV, such as 3′-(3-methylbut-2-enyl)-3′,4,7-trihydroxyflavane, papyriflavonol A (Park et al. 2017), herbacetin, quercetin 3-*O*-β-D-glucoside and isobavachalcone (Jo et al. 2019). Other instances are resveratrol (Lin et al. 2017) and conessine (Shen et al. 2019).

### Avian infectious bronchitis virus (IBV)

IBV evokes in unvaccinated chicken and turkey bronchitis, cough, fever, conjunctivitis and fatal respiratory distress. The spike proteins bind to sialic acid and the virus is internalised to release a single-stranded (+)-RNA that is transcribed into structural proteins and enzymes. Perusal of literature indicates that natural products in medicinal plants in Asia and the Pacific are meek repressors of IBV in general such as forsythoside A (Li et al. 2011) or β-pinene (Yang et al. 2011).

### Dengue virus (DV)

DV is responsible for fever and bleeding. The virion binds via the envelop protein E to heparan sulphate on the surface of host cells (Chen et al. 1997). Very strong inhibitors of DV *in vitro* are Amaryllidaceae alkaloids (Table 1).

### Simple phenolics and hydroxycinnamic acid derivatives

The benzofuran quinone usnic acid from members of the genus *Diospyros* L. (Ebenaceae) restrained DV by 90% at the concentration of 5 µg/mL (Peyrat et al. 2016). Other examples are salidroside (Sharma et al. 2016), tatanan A (Yao et al. 2018) and flacourtiaceous phenolic glycosides (Bourjot et al. 2012).

### Flavonoids

Morin and baicalein ($IC_{50}$ 5.3 µg/mL) are strong DV suppressors (Maharani et al. 2020). Other examples are pectolinarin (Simões et al. 2011) and quercetrin (Chiow et al. 2016).

### Lignans

The neolignan honokiol from *Magnolia officinalis* Rehder and E.H. Wilson (Magnoliaceae) is a strong repressor of DV (Fang et al. 2015).

### Alkaloids

Amaryllidaceae alkaloids from *C. asiaticum* are very strong inhibitors of DV *in vitro*, such as pseudolycorine (**23**), lycoricidine (**24**) ($IC_{50}$ 0.005 µg/mL) and pancratistatin (**25**) ($IC_{50}$ 0.06 µg/mL) (Gabrielsen et al. 1992). Moderate suppressors are palmatine, vincosamide, hirsutine (**100**) (Jia et al. 2010; Costa et al. 2020) and voacangine (Gómez-Calderón et al. 2017).

### Triterpenes and saponins

Betulinic acid, (3β)-3.23-dihydroxylup-20(29)-en-28-oic acid, (3β)-3-(acetyloxy)-urs-12-en-28-oic acid, betulinic aldehyde and (3β)-3.23-dihydroxylup-12.20(29)-dien-28-oic acid at the concentration of 10 µg/mL inhibited DV by about 50%, (Peyrat et al. 2016). Celastrol from *Tripterygium wilfordii* Hook. f. (Celastraceae) strongly restrained DV ($IC_{50}$ 0.1 µM) (Yu et al.

2017) and was active *in vivo* (Yu et al. 2017). Meliaceae yield strong DV repressors, such as the limonoids swielimonoid B, swietenolide, swietenine acetate and methyl angolensate (*Swietenia macrophylla* King) (Cheng et al. 2014) and the tirucallane triterpenes 3-α-tigloyl-melianol and melianone (*Melia azedarach* L.) (Sanna et al. 2015). Glycyrrhizin is weakly active (Crance et al. 2003).

### Japanese encephalitis virus (JEV)

JEV accounts for fatal central nervous system damage. The virion E protein binds to the host cell LDL-receptor and the rest of the cycle is similar to as DV (Yun and Lee 2014). Very strong repressors of JEV are Amaryllidaceae alkaloids (Table 1).

#### Phenolics

Phenolics are often strong JEV inhibitors *in vitro*, such as the flavanol hesperitin (**19**) in *Citrus sinensis* (L.) Osbeck (Rutaceae) ($IC_{50}$ 3.6 µg/mL; S.I. >159) (Lin et al. 2008) and baicalein ($IC_{50}$ 14.2 µg/mL) (Johari et al. 2012). Other examples are aloe-emodin (**2**) in *Hemerocallis fulva* (L.) L. (Asphodelaceae) ($IC_{50}$ 1.5 µg/mL; S.I. >200) (Lin et al. 2008), 2-(1- hydroxyethyl)naphtho[2,3-b]furan-4,9-dione in *Kigelia pinnata* (Jacq.) DC. (Bignoniaceae) (Takegami et al. 1998) and shikonin from *Lithospermum erythrorhizon* Siebold and Zucc. (Boraginaceae) ($IC_{50}$ 2.1 µg/mL) (Lin et al. 2008). Active intraperitoneally against JEV are arctigenin from *Arctium lappa* L. (Asteraceae) and the hydroxycinnamic acid derivative rosmarinic acid in Lamiaceae plants (Swarup et al. 2008).

#### Alkaloids

Amaryllidaceae alkaloids are very strong suppressors of JEV such as narciclasine (**26**) ($IC_{50}$ 0.001 µg/mL), lycoricidine (**24**) ($IC_{50}$ 0.005 µg/mL), pseudolycorine (**23**) ($IC_{50}$ 0.2 µg/mL) and pretazettine (**27**) ($IC_{50}$ 0.6 µg/mL) (Gabrielsen et al. 1992). Of note, pancratistatin (**25**) ($IC_{50}$ 0.01 µg/mL) given subcutaneously at the dose of 4 mg/kg/d once daily for 7 d evoked 100% protection in mice (Gabrielsen et al. 1992). The bisbenzylisoquinoline berbamine given intraperitoneally at the dose of 15 mg/kg, twice per day to mice evoked a survival rate of 75% (Huang et al. 2021). Other inhibitors are arecoline (Lin et al. 2008), atropine (Yamazaki and Tagaya 1980) and scopolamine (Bhattacharjee et al. 2021).

### Yellow fever virus (YFV)

YFV is transmitted by mosquitoes and produces in unvaccinated people fever, jaundice, myalgia and in some individual fatalities. The virion surface E glycoprotein binds to host cells *via* an unidentified receptor and the viral cycle is identical with DV (Gardner and Ryman 2010). Natural products inhibiting DV tend to be active against YFV, such as melianone ($EC_{50}$ 3 µM) (Sanna et al. 2015), naringenin (**28**) and hesperitin (**29**) ($EC_{50}$ 0.001 and 0.01 µM, respectively) (Castrillo et al. 2015). Examples of very strong YFV inhibitors are narciclasine (**26**), lycoricidine (**24**), pseudolycorine (**23**), pancrastitatin (**11**) and pretazettine (**27**) with $IC_{50}$ values of 0.006, 0.005, 0.3, 0.01 and 0.5 µg/mL, respectively (Gabrielsen et al. 1992), as well as palmatine ($IC_{50}$ 0.5 µg/mL) (Gabrielsen et al. 1992).

### West Nile virus (WNV)

WNV is transmitted by mosquitoes and responsible for fever, rashes and fatal neurological damages globally (Campbell et al. 2002). Lycorine (**3**) inhibited WNV (epidemic strain) with an $IC_{50}$ value of 0.2 µM (Zou et al. 2009). 3-α-Tigloyl-melianol is a strong WNV suppressor ($EC_{50}$ 3 µM) (Sanna et al. 2015) whereas glycyrrhizin is weakly active (Crance et al. 2003). Other instances are hesperetin, hesperidin (Eberle et al. 2021) and palmatine (Jia et al. 2010).

### Zika virus (ZIKV)

ZIKV is a zoonotic virus transmitted by mosquitoes and responsible for outbreaks of fever, rashes, conjunctivitis, foetal anomalies, myalgia and in some patients sever neurological disorder. The virion surface protein E binds to lectins and after internalisation, (+)-RNA is translated into a polyprotein cleaved by viral protease complex NS2B-NS3 (Sager et al. 2018).

#### Phenolics

The strongest inhibitors of ZIKV within the phenolics group are lignans, such as silvestrol (**8**) ($IC_{50}$ 10 nM) (Elgner et al. 2018) and diphyllin (**30**) from *Justicia gendarussa* L. (Acanthaceae) ($IC_{50}$ 0.06 µM) (Cui et al. 2014). Flavonoids are moderate suppressors of ZIKV replication *in vitro* such as naringenin (**28**) (Cataneo et al. 2019), isoquercitrin (Gaudry et al. 2018), pinocembrin (Le Lee et al. 2019), hesperetin, myricetin, luteolin and pedalitin (Cataneo et al. 2019; Lima et al. 2021). Other phenolics with moderate potencies are curcumin ($IC_{50}$ 1.9 µM) (Mounce et al. 2017) and crenatoside (Reis et al. 2020).

#### Alkaloids

Hippeastrine from *Lycoris radiata* (L′ Hér.) Herb. (Amaryllidaceae) inhibited ZIKV with an $IC_{50}$ value of 1.9 µM and given subcutaneously at a dose of 100 mg/kg/d to mice evoked some levels of protection (Zhou et al. 2017). Palmatine and glycyrrhizin are weakly active (Crance et al. 2003).

#### Terpenes

Cardiac glycosides, such as ouabain and digitoxin are broad-spectrum antiviral molecules acting on the replication of CHIKV, MERS and SARS-CoV (Amarelle and Lecuona 2018; Yang et al. 2018). Conessine is active ($IC_{50}$ 9.7 µM) (Lima et al. 2021).

### Hepatitis C virus (HCV)

HCV is transmitted by blood via transfusion or sex and account for hepatocellular carcinoma in humans. The virion surface glycoprotein E binds to heparan sulphate of hepatocytes and (+)-RNA is translated into a polyprotein cleaved into structural proteins and enzymes, such as HCV NS5B polymerase (Bhatia et al. 2014).

#### Simple phenolics

Gallic acid (**1**) (Hsu et al. 2015) and ellagic are moderate HCV inhibitors (Ajala et al. 2014) as well as methylgallate, bergenin and ethyl gallate in *Saxifraga melanocentra* Franch. (Saxifragaceae) (Zuo et al. 2005).

### Coumarins

The furanocoumarin chalepin from *Ruta angustifolia* (L.) Pers. (Rutaceae) strongly inhibited HCV with the $IC_{50}$ value of 1.7 µg/mL and the S.I. of 8.2 (Wahyun et al. 2014). Wedelolactone is a moderate HCV repressor (Manvar et al. 2012) as well as alloxanthoxyletin from *Melicope latifolia* (DC.) T.G. Hartley (Rutaceae) (Widyawaruyanti et al. 2021).

### Flavonoids

Robinetin in *Adenanthera pavonina* L. (Fabaceae) (Ahmed-Belkacem et al. 2014), rutin (**92**) in *Sophora japonica* L. (Fabaceae) (Zuo et al. 2005), kaempferol-3,7-bisrhamnosidein *Taxillus sutchuenensis* (Lecomte) Danser (Loranthaceae) (Yang et al. 2017), sorbifolin, pedalitin from *Ruellia tuberosa* L. (Acanthaceae) (Shimizu et al. 2017), quercetin, kaempferol and kaempferol-3-β-D-glucoside (Zuo et al. 2005) are modest inhibitors.

### Lignans

*Trachelospermum jasminoides* (Lindl.) Lem. (Apocynaceae) yields the dibenzylbutyrolactone lignan trachelogenin (**31**) that very strongly inhibited HCV ($IC_{50}$ 0.3 µg/mL) (Qian et al. 2016). Another instance is 3-hydroxy caruilignan C from *Swietenia mahagoni* (L.) Jacq. (Meliaceae) (Wu, Cheng, et al. 2012; Wu, Lin, et al. 2012).

### Stilbenes

The oligostilbenoid ε-viniferin repressed HCV with the $EC_{50}$ value of 0.1 µM (Lee et al. 2019).

### Naphthoquinones

Plumbagin (**32**) in *Plumbago indica* L. (Plumbaginaceae) very strongly suppressed HVC ($IC_{50}$ 0.5 µg/mL; S.I. 53.7).

### Tannins

Hydrolysable tannins inhibit HCV *in vitro*, such as 1,2,3,4,6-penta-*O*-galloyl-β-D-glucoside and 1,3,4,6-tetra-*O*-galloyl-β-D-glucoside in *S. melanocentra* (Zuo et al. 2005), 1,2,3,6-tetragalloy1-β-D-glucopyranose from *C. officinalis*, pentagalloyl glucose (Ajala et al. 2014), tellimagrandin I and II (Yue et al. 2006), casuarinin, corilagin and chebulagic acid from *Terminalia chebula* Retz. (Combretaceae) (Ajala et al. 2014).

### Alkaloids

Rutaceous alkaloids are strong suppressors of HCV such as kokusaginine, arborinine and pseudane IX with the $IC_{50}$ values of 6.4, 6.4 and 1.4 µg/mL, respectively (Wahyun et al. 2014). Another example is *N*-methylflindersine from *M. latifolia* ($IC_{50}$ 3.8 µg/mL) (Widyawaruyanti et al. 2021).

### Terpenes

The iridoid glycoside loganic acid (*C. officinalis*) is a strong HCV suppressor (Zhang et al. 2009) as well as the lindenane sesquiterpenes chlorajaponilide F, sarcandrolide F and shizukaol E from *Chloranthus japonicus* Siebold (Chloranthaceae) (Yan et al. 2016), and the sesquiterpene lactones cynaropicrin in *Cynara scolymus* L. (Asteraceae) ($EC_{50}$ 1 µM) (Elsebai et al. 2016). Other examples are the spirostane steroid diosgenin in *Dioscorea batatas* Decne. (Dioscoreaceae) (Wan et al. 2011), and the oleanane triterpene saponins platycodin D (**33**), D2 and D3 from *Platycodon grandiflorus* (Jacq.) A. DC. (Campanulaceae) with the $IC_{50}$ values of 0.3, 1 and 2.4 µg/mL, respectively (Kim et al. 2013).

### Miscellaneous

Lucidone from *Houttuynia cordata* Thunb. (Saururaceae) (Chen et al. 2013) and loliolide from *Phyllanthus niruri* L. (Phyllanthaceae) ($EC_{50}$ 2.4 µg/mL; S.I. 62.6) (Chung et al. 2016).

### Chikungunya virus (CHIKV)

CHIKV is zoonotic, transmitted by mosquitoes and responsible for fever, rashes, nausea, join pains and myalgia that can last for years (Deeba et al. 2016). The virion binds to host cell *via* its surface glycoprotein E, and viral (+)-RNA is translated into a polyprotein that is cleaved by viral proteases into structural proteins and enzymes (Deeba et al. 2016). Phenolics strongly inhibit CHIKV *in vitro* such as curcumin ($IC_{50}$ 3.8 µM) (Mounce et al. 2017), resveratrol (Lin et al. 2017), pinocembrin (Le Lee et al. 2019), baicalein ($IC_{50}$ 1.8 µg/mL; S.I. 188.4) (Lani et al. 2016), silvestrol (**8**) (5 nM) (Henss et al. 2018) and epigallocatechin-3-gallate (Weber et al. 2015). In the case of alkaloids, berberine (**97**) strongly restrained CHIKV (Varghese et al. 2016). Euphorbiaceae plants yield tigliane diterpenes with strong activity such 12-*O*-tetradecanoylphorbol 13-acetate (**34**) ($EC_{50}$ 2.9 nM; S.I. >1000), prostatin, 4α-12-*O*-tetradecanoylphorbol 13-acetate (**34**) (Bourjot et al. 2012), and 12-*O*-decanoyl-7-hydroperoxyphorbol-5-ene-13-acetate (Corlay et al. 2014). Other examples are daphnane diterpenes, such as trigocherrierin A and trigocherriolide E from *Trigonostemon cherrieri* J.M Veillon (Bourjot et al. 2014).

### Sindbis virus (SINV)

In the Euphorbiaceae, the tigliane diterpene 12-*O*-tetradecanoylphorbol 13-acetate (**34**) inhibited SINV with the $EC_{50}$ value of 2.2 µM and a S.I. above 162 (Bourjot et al. 2012).

### Mayaro virus (MAYV)

MAYV is transmitted from primates to humans via mosquitoes and accounts for rashes, fever, myalgia, retro-orbital pain, headache, diarrhoea and long-lasting arthralgia (Esposito et al. 2016). The virion binds to heparan sulphate to release a (+)-RNA that is directly translated by host ribosome into a polyprotein later cleaved into functional proteins (Mota et al. 2015). Epicatechin (**35**) strongly repressed MAYV (strain Bear 20290) with the $IC_{50}$ value of 0.2 µM (Ferraz et al. 2019).

### Semliki Forest virus (SFV)

SFV is transmitted by mosquitoes and responsible for fever, myalgia and arthralgia. The virus follows standard α-coronavirus cell cycle. The iridoid glycoside arbortristoside A given orally at the dose of 62.5 mg/kg to rodents of evoked some levels of protection. In the Lamiids, *Heliotropium subulatum* Hochst. ex DC. (Boraginaceae) yields the hydroxycinnamic acid derivative subulacine that strongly suppressed SVF (Singh et al. 2002). *ent*-14*S**-hydroxykaur-16-en-19-oic acid in the genus *Croton* L. (Euphorbiaceae) (Langat et al. 2012) and the quassinoid simalikalactone in the genus *Quassia* L. (Simaroubaceae) are moderate SFV inhibitors (Apers et al. 2002). Lycorine (**3**) is strongly active.

## Human immunodeficiency virus (HIV)

HIV is a zoonotic virus transmitted with blood from apes to humans and responsible for AIDS (De Clercq 2007). This virus target CD4 cells resulting in a decrease of immunity, which in most untreated patient, leads to fatality. The viral glycoprotein 120 binds to the CCR5 and CXCR4 receptor and viral RNA is translated to DNA by viral reverse transcriptase (Zack 1995). A broad array of natural products is able to repress HIV *in vitro* (Table 1).

### Simple phenolics

Hydroxytyrosol (**36**) from *Sargentodoxa cuneata* (Oliv.) Rehder and E.H. Wilson (Lardizabalaceae) very strongly restrained HIV ($IC_{50}$ 61 nM; S.I. >1000) (Lee-Huang et al. 2007). Rottlerin ($IC_{50}$ 2.2 µM) from *Mallotus philippensis* (Lam.) Müll. Arg. (Euphorbiaceae) (López-Huertas, et al. 2011), protocatechuic acid, norbergenin, bergenin (Piacente et al. 1996), 5,7-dimethoxyphthalide (Lin et al. 2010), garcimangosone D (Wu, Cheng, et al. 2012; Wu, Lin, et al. 2012), 2-methoxy-2-butenolide-3-cinnamate (Said et al. 2021), ellagic acid (Modi et al. 2013), garciosine B (Pailee et al. 2018) and pellucidin A (Thongphichai et al. 2019) are moderately active.

### Hydroxycinnamic acid derivatives

These are strong to very strong HIV repressors *in vitro*, such as 4,5-di-*O*-caffeoylquinic acid (**40**) from *Guettarda speciosa* L. (Rubiaceae) ($EC_{50}$ 0.6 µg/mL) (Mahmood et al. 1993) and 1′*S*-1′-acetoxychavicol acetate from *Alpinia galanga* (L.) Willd. (Zingiberaceae) (Ye and Li 2006). Other examples include, lithospermic acid in *Tournefortia sarmentosa* Lam (Boraginaceae) (Bailly and Cotelle 2005), as well as niruriside (*P. niruri*) (Qian-Cutrone et al. 1996).

### Flavonoids

Taxifolin from *Polygonum capitatum* Buch.-Ham. ex D. Don (Polygonaceae) (Min et al. 2002), catechin (Lin et al. 2010), gallocatechin, (-)-epicatechin-3-*O*-benzoate (Wu, Cheng, et al. 2012; Wu, Lin, et al. 2012), dihydrokaempferol (Wang et al. 2014), pinocembrin (Wang et al. 2014), as well as the flavan glycoside homoeriodictyol-7-*O*-β-D-glucopyranoside-4′-*O*-β-D-apiofuranoside (Szurpnicka et al. 2020) are moderately active. Introduction of a ketone group in position 3 of flavans increases the anti-HIV activity and this is exemplified with kuwanon L from the genus *Morus* L. in the Moraceae ($EC_{50}$ 1.9 µM) (Esposito et al. 2015). Flavonols are mainly strong inhibitors of HIV-1 *in vitro* as seen with (-)-5,4″-dihydroxy-7,8-[(3″-hydroxy-4″-one)-2″,2″-dimethylpyrano]-flavone ($EC_{50}$ 0.9 µM) identified from *Citrus trifoliata* L. (Rutaceae) (Feng et al. 2010) or tricin (Poaceae) (Matsuta et al. 2011). Other HIV repressors are baicalein, scutellarein (Ono et al. 1990), apigenin 7-*O*-β-D-(4″-caffeoyl)glucuronide (Lee et al. 2003), orobol, calycosin in *Astragalus mongholicus* Bunge (Fabaceae) ($EC_{50}$ 2.2 µg/mL) (Chen et al. 2011), biochanin A (Lin et al. 2007), quercetin 3-*O*-(6″-feruloyl)-β-D-galactopyranoside (Datta et al. 2004), kaempferol-7-*O*-glucoside, and dihydrokaempferol-3-*O*-rhamnoside (Wang et al. 2014). Quercetin 3-*O*-β-D-glucuronide from *Nelumbo nucifera* Gaertn. (Nelumboaceae) is strongly active ($EC_{50}$ 2 µg/mL; S.I. >20) (Kashiwada et al. 2005). The dimerisation of flavones yields antiretroviral bisflavones, such as hinokiflavone, agathisflavone, morelloflavone (Lin et al. 1997) and chamaeflavone A. Within the Fabaceae, *Cassia siamea* Lam. yields the chromone siamchromone D that strongly suppressed HIV-1 ($IC_{50}$ 1.8 µg/mL; S.I. >100) (Hu et al. 2012).

### Coumarins

Clusiaceae plants yield prenylated coumarins with strong antiretroviral activities *in vitro*. For instance, calophyllum B and P from *Calophyllum inophyllum* L. (Patil et al. 1993) or calanolide A (**41**) from the Sarawakian rainforest tree *Calophyllum teysmannii* var. *inophyloides* (King) P.F. Stevens ($IC_{50}$ 0.1 µM; S.I. 40) (Galinis et al. 1996). Mesuol from *Mesua ferrea* L. has much lower potencies (Márquez et al. 2005). In the Rutaceae, the pyranocoumarin clausenidin (*Clausena excavata* Burm.f.) (Sunthitikawinsakul et al. 2003) and the furancoumarin aurapten (Sabde et al. 2011) are moderately active whereas imperatonin from *Aegle marmelos* (L.) Corrêa is a strong inhibitor (Sabde et al. 2011). Isopimpinellin (**38**) and *O*-methylcedrelopsin (**39**) from *Zanthoxyllum ailanthoides* Siebold and Zucc (Rutaceae) restrained HIV-1 (IIIB strain) with $EC_{50}$ values below 0.6 and 0.5 µg/mL, respectively (Cheng et al. 2005). Wedelolactone displayed some levels of antiretroviral effects.

### Naphthoquinones

The pyranonaphthoquinone isoeleutherin from *Eleutherine americana* (Aubl.) Merr. ex K. Heyne (Iridaceae) suppressed HIV with the $IC_{50}$ value of 8.5 µg/mL (Insanu et al. 2014). Another instance is juglone (Min et al. 2002).

### Xanthones

Caged prenylated xanthones in the genus *Garcinia* L. (Clusiaceae) are strong repressors of HIV *in vitro* but have low S.I. This is the case for hanburin and dihydroisomorellin with the $EC_{50}$ of 3 and 1.2 µg/mL and S.I. of 1.7 and 4, respectively) (Reutrakul et al. 2006). Other instances are mangiferin and 1,3,5-trihydroxyxanthone (Pailee et al. 2018). In the family Rubiaceae, xanthopurpurin (Sabde et al. 2011), and in the Polygonaceae sennosides A and B (*Rheum palmatum* L.) chrysophanol and emodin-8-*O*-β-D-glucopyranoside are antiretroviral (Esposito et al. 2016).

### Lignans

Lignans and norlignans, and especially from basal Clades, very strongly restrain HIV and HIV enzymes *in vitro*. Examples are hinokinin (**42**) from *Piper cubeba* L. (Piperaceae) ($IC_{50}$ <1 µg/mL) (Cheng et al. 2005), the dibenzocyclooctadiene lignans rubrilignan B ($EC_{50}$ 1.8 µg/mL; S.I. 18.6) from *Schisandra rubriflora* (Franch.) Rehder and E.H. Wilson (Schisandraceae) (Mu et al. 2011), wilsonilignan C ($EC_{50}$ 2.8 µg/mL) and schilancifolignan A ($EC_{50}$ 2.3 µg/mL) from *Schisandra bicolour* W.C. Cheng (Yang, Lee, et al. 2010; Yang, Li, et al. 2010), gomisin M1 (Chen et al. 2006), binankadsurin A (Gao et al. 2008), tiegusanin G (Li et al. 2009), wulignan A2 (Xiao et al. 2010) and marlignan L (Yang, Lee, et al. 2010; Yang, Li, et al. 2010). *J. gendarussa* yields the arylnaphthalene lignan glycoside justiprocumin B (**37**) that repressed HIV with the $IC_{50}$ value 15 nM (Zhang, Ai, et al. 2017; Zhang, Rumschlag-Booms, et al. 2017). The tetrahydrofuran lignans kadlongirin A and B (Pu et al. 2008), manassantin A and B (Lee et al. 2010), demethoxyepiexcelsin (Hoang et al. 2002; +)-(7′*S*,8′*R*)-erythro-7′-methylcarolignan E (Jiang et al. 2016), nyasol (Zhang et al. 2004) and anolignan A (Rimando et al. 1994) are moderately to weakly active.

## Stilbenes

Resveratrol is antiretroviral ($EC_{50}$ 4.3 µg/mL) (Lin et al. 2010). Of lesser strength are oxyresveratrol (Likhitwitayawuid et al. 2005) and 3-hydroxy-5-methoxystilbene (Said et al. 2021). Scirpusin B from the aerial parts of *Caragana rosea* Turcz. ex Maxim. (Fabaceae) is strongly active ($EC_{50}$: 7 µg/mL) (Yang et al. 2005).

## Tannins

Plants in the Clade Fabids yield gallotannins that repress HIV, such as tetragalloyl quinic in *Galphimia glauca* Cav. (Malpighiaceae) (Nishizawa et al. 1989), 1,2,6-trigalloylglucopyranose and 1,2,3,6-tetragalloyl glucopyranose from *Juglans mandshurica* Maxim. (Juglandaceae) (Min et al. 2004). Other examples are 1,3,6-tri-*O*-galloyl-β-D-glucopyranose and 1,2,3,4,6-penta-*O*-galloyl-β-D-glucopyranose (Ahn et al. 2002). The ellagitannin geraniin (**43**) from *Phyllanthus amarus* Schumach. and Thonn. (Phyllanthaceae) ($EC_{50}$: 0.4 µM; S.I. 29.7 (Notka et al. 2003), as well as camelliatannin H from *C. japonica* L ($IC_{50}$ 0.9 µM), and chebulagic acid (Ajala et al. 2014) are active (Park et al. 2002), as well as the condensed tannin proanthocyanidin A2 (Suedee et al. 2013; Shahat et al. 1998).

## Alkaloids

Isoquinolines from basal Clades have strong to very strong anti-HIV activities *in vitro*, such as coclaurine (**44**), liensinine (**45**), neferine (**29**) and isoliensinine (**47**) from *N. nucifera* ($EC_{50}$ 0.8 µg/mL; S.I.>20) (Kashiwada et al. 2005), as well as cycleanine from *Cissampelos hirsuta* Buch.-Ham. ex DC. (Menispermaceae) ($IC_{50}$ 1.8 µg/mL: S.I. 9) (Otshudi et al. 2005). Another example is fangchinoline (**21**) from *Stephania japonica* (Thunb.) Miers (Menispermaceae) ($EC_{50}$ 0.8 µM) (Wan et al. 2012). From this climber, the aporphine alkaloid nornuciferine (**48**) very strongly restrained HIV ($EC_{50}$<0.8 µg/mL; S.I. 20) (Kashiwada et al. 2005). Other examples are lycorine (**3**) (Szlávik et al. 2004), berberine (**98**) and protopine (Sabde et al. 2011). Plants in the genus *Ancistrocladus* Wall. (Ancistrocladaceae) generate unique series of antiretroviral isoquinolines oligomers, such as korundamine A (Hallock et al. 1998), michellamine $A_2$, $A_3$ and $A_4$ (Bringmann et al. 2016). Indole alkaloids are often strongly active such as indole-3-carboxylic acid from *Begonia nantoensis* M.J. Lai and N.J. Chung ($EC_{50}$ 2.4 µg/mL) (Wu et al. 2004) or (*E*)-3-(3-hydroxymethyl-2-butenyl)-7-(3-methyl-2-butenyl)-1H-indole in *Glycosmis montana* Pierre (Rutaceae) ($IC_{50}$ 1.1 µg/mL; S.I. 11.6) (Wang et al. 2005). The β-carboline alkaloid drymaritine (**49**) from *Drymaria cordata* (L.) Willd. ex Schult. (Caryophyllaceae) is very strongly antiretroviral ($EC_{50}$ 0.6 µg/mL; S.I. 20.6) (Hsieh et al. 2004).

As for phenanthroindolizidine alkaloids, dehydroantofine identified from *Cryptocarya chinensis* (Hance) Hemsl. (Lauraceae) strongly restrained HIV-1 ($EC_{50}$ 1.8 µg/mL) (Wu, Cheng, et al. 2012; Wu, Lin, et al. 2012). Celastraceae plants yield unique types of sesquiterpene pyridine alkaloids with very strong anti-HIV activities (such as wilfortrine (**50**), wilfordin (**51**), hypoglaunine A (**52**) and B (**53**) $EC_{50}$ 0.1 µg/mL) (Horiuch et al. 2006). Other anti-HIV alkaloids in the Clade Fabids are indolizidine oligomers including fluevirosinine B and virosecurinine (Zhang, Wu, et al. 2015; Zhang, Zhang, et al. 2015). *Melochia odorata* L.f. (Malvaceae) produces the epoxide quinolinone alkaloid waltherione C that yielded the $EC_{50}$ value of 0.8 µM (Jadulco et al. 2014). Rutaceous benzophenanthridine, quinoline, carbazoles and amide alkaloids are often very strong suppressors of HIV, such as and 6-acetonyldihydronitidine (**54**), decarine (**55**), dihydroavicine (**56**), haplopine (**57**), 4-methoxy-1-methyl-2-quinolone (**58**), *O*-methyltembamide (**59**), oxyavicine (**60**) and (+)-tembamide (**61**) ($EC_{50}$ <1 µg/mL) (Cheng et al. 2005). Other instances are (+)-*S*-deoxydihydroglyparvin (Chansriniyom et al. 2009) and *O*-methylmukonal (Sunthitikawinsakul et al. 2003).

## Terpenes

Examples are zerumbone (Dai et al. 1997), verticillatol (Hoang et al. 2002), tashironine A (Song et al. 2007), chlorajaponilides F and G, sarcandrolide F and shizukaol E (Yan et al. 2016). The lindenane sesquiterpene shizukaol B from *C. japonicus* ($EC_{50}$ 0.2 µM) and the cadinane sesquiterpene 2,7-dihydroxy-11,12-dehydrocalamenene ($EC_{50}$ 12.8 µg/mL) in *Kadsura longipedunculata* Finet and Gagnep. (Schisandraceae) (Pu et al. 2008) are strongly active. Other antiretroviral sesquiterpenes are the cadinane dimer gossypol from *Gossypium arboreum* L. (Malvaceae) ($IC_{50}$ 0.6 µM) (Polsky et al. 1989) as well as the guaiane sesquiterpene viscoazulone (Datta et al. 2004).

Diterpenes in general are strong inhibitors of HIV including 16β-17-dihydroxy-*ent*-kauran-19-oic acid (**62**) from *Annona squamosa* L. (Annonaceae) ($EC_{50}$ 0.8 µg/mL; S.I. >5) (Rasool et al. 2005). Another instance is the labdane andrographolide from *A. paniculata* (Gurrapu and Mamidala 2017) which given orally to AIDS patients evoked a mean increase in $CD4^{+}$ cell counts (Calabrese et al. 2000). In the Lamiids, cembrane diterpenes such as ovatodiolide curbed HIV (Alam et al. 2000). *T. wilfordii* produces the very strong antiretroviral abietane diterpene triptolide (**63**) ($EC_{50}$ 0.3 nM) (Wan and Chen 2014). Euphorbiaceae and Thymeleaceae plants engineer very strong antiretrovial tiglianes such as excoecafolin B (**64**) and C (**65**) (*Excoecaria acerifolia* Didr.) ($EC_{50}$ 0.03 and 0.04 µM; S.I. 431.1 and 298.7) (Huang et al. 2014), 12-deoxyphorbol 13-(3*E*,5*E*)-decadienoate (**66**) (*Excoecaria agallocha* L.) ($IC_{50}$ 6 nM) (Erickson et al. 1995), stelleralide A (**67**) ($EC_{90}$ 0.4 nM) and stelleracin B (**68**) and C (**69**) (*Stellera chamaejasme* L.) ($EC_{90}$ 0.0006 and 0.003 µM). The daphnane diterpene trigonothyrin F (**70**) from *Trigonostemon thyrsoideus* Stapf (Thymeleaceae) yielded the $IC_{50}$ value of 0.1 µg/mL and a S.I. of 75.1. Other examples are trigolin C, trigolin G and trigochinin A (Li et al. 2013).

Within the oleanane triterpenes, moronic acid (**71**) in *R. javanica* ($IC_{50}$ < 0.1 µg/mL; S.I. >186) (Ito et al. 2001) and oleanolic acid ($EC_{50}$ 1.7 µg/mL) (Kashiwada et al. 1998) are strongly antiretroviral. Other examples are 3β,22α-dihydroxyolean-12-en-29-oic acid (Wu et al. 2004), 3-oxo-olean-12-en-29-oic acid, katonic acid, koetjapic acid (Sun et al. 1999), papyriogenin A (Kuo et al. 2009) and 21β-*O*-[(2*E*)-6-hydroxyl-2,6-dimethyl-2,7-octadienoyl] pitheduloside G (Nguyen et al. 2018). The ursane triterpene pomolic acid in *Hyptis capitata* Jacq. (Lamiaceae) inhibited HIV with an $EC_{50}$ value of 1.4 µg/mL and S.I. of 16.6 (Kashiwada et al. 1998). As for lupanes, dammaranes, lanostanes and cycloartanes, they are moderately to strongly active, such as 2-acetoxyalphitolic acid, 3-acetoxyalphitolic acid, betulinic acid (Reutrakul et al. 2010), garciosaterpene A ($EC_{50}$ 5.8 µg/mL) and D, ocotillone (Chang et al. 2003) and dammarenolic acid (Esimone et al. 2010). Other examples are dammara-20,25-dien-3β,24-diol ($IC_{50}$ 2.7 µg/mL) from the genus *Vatica* L. (Dipterocarpaceae) (Zhang et al. 2003), suberosol from *Polyalthia suberosa* (Roxb.) Thwaite (Annonaceae) ($EC_{50}$ 3 µg/mL) (Li et al. 1993; 24*E*)-3-oxo-lanostane-8,24-dien-26-oic acid and angustific acid A (Sun et al. 2011). Limonoids are often very strongly antiretroviral such as clausenolide-1-ethyl ether (**76**) from *C. excavata* ($EC_{50}$ $3.4 \times 10^{-5}$ M; S.I. 16) (Sunthitikawinsakul et al. 2003) and limonin (**72**) from *Citrus × bergamia* Risso and Poit (Rutaceae) ($IC_{50}$ 0.9 µg/mL) (Battinelli et al. 2003). Other anti-HIV limonoids are nomilin (Battinelli et al. 2003), sundarbanxylogranin B (Dai et al. 2017), ciparasin B (Yu et al. 2015), trichiconin B (Liu et al. 2014) and azadirachtin (David et al. 2017).

The oleanane saponin arganine (Gosse et al. 2002), the cycloartane saponin actein (**73**) from *Cimicifuga foetida* L. (Ranunculaceae) ($EC_{50}$ 0.3 µg/mL; S.I. of 144), oleandrin (**74**) (50–200 ng/ml) (Singh et al. 2013), glycyrrhizin (Harada 2005) and asiaticoside (Jaisi et al. 2021) are active.

#### Miscellaneous

Biyouyanagin A (**75**) in *Hypericum japonicum* Thunb. (Hypericaceae) ($EC_{50}$ 0.7 µg/mL; S.I. above 31.1) (Tanaka et al. 2005), the polyacelylene fatty acid minquartynoic acid in *Onachanostachys amentacea* Mast. (Olacaceae) ($EC_{50}$ 2 µg/mL) (Rashid et al. 2001), 5-hydroxymethyl-(2,2′:5′,2″)-terthienyl tiglate, 3-epilitsenolide D2 (Zhang et al. 2005), litseaverticillol B ($IC_{50}$ 3 µg/mL) in *Litsea verticillata* Hance (Lauraceae) (Zhang et al. 2003) and isolitseane B (Zhang et al. 2005) are antiretroviral.

## (−) RNA viruses

### Ebola virus (EBOV)

EBOV is zoonotic from bats, apes and pigs and responsible in Africa and the Philippines for haemorrhagic fever with a fatality rate of about 50%. The virion binds to the DC-SIGN receptor of host cell and (-)- RNA is transcripted by the viral polymerase complex (Hoenen et al. 2012). Strong phenolic inhibitors of EBOV are ellagic acid ($IC_{50}$ 1.4 µM) (Cui et al. 2018), eugenol ($EC_{50}$ 1.3 µM) (Lane et al. 2019) and silvestrol (**8**) (10 nM) (Elgner et al. 2018). Oleandrin (**74**) suppressed EBOV with a $IC_{50}$ value of 0.02 µM (James et al. 2017).

### Newcastle disease virus (NDV)

NDV incurs fever, diarrhoea, anorexia and fatality in unvaccinated poultry. The surface haemagglutinin–neuraminidase glycoprotein of the virion binds to host cell sialic acid and the single-stranded (-)-RNA is translated by the viral RNA-dependent RNA polymerase into a (+)-RNA used for the synthesis of proteins by host ribosomes (Zaitsev et al. 2004). Hydroxytyrosol (**36**) (Yamada et al. 2009) and glycyrrhizin are moderate repressors of NDV.

### Measles virus (MEV)

MEV is fatal for unvaccinated children especially in developing countries. The virion binds via its surface haemagglutinin–neuraminidase glycoprotein to CD46 or PVRL4 host cell receptors and after capsid internalisation the single-stranded (-)-RNA is translated by viral RNA-dependent RNA polymerase into a (+)-RNA used for the synthesis of protein by host ribosomes (Bhattacharjee and Yadava 2018). The naphthoquinone droserone in *Drosera peltata* Thunb. (Droseraceae) is moderately active against this virus (Lieberherr et al. 2017) as well as angeloyl heliotrine (Singh et al. 2002) and the dammarane triterpene saponin chikusetsusaponin IV a from *Alternanthera philoxeroides* (Mart.) Griseb. (Amaranthaceae) (Rattanathongkom et al. 2009).

### Parainfluenza-3 virus (HPIV-3)

HPIV-3 binds *via* its surface haemagglutinin–neuraminidase glycoprotein to the host cell sialic acid and the single-stranded (-)-RNA is released in the cytoplasm and copied to a (+)-RNA *via* RNA dependent RNA (Lawrence et al. 2004). Flavones are moderate suppressors of HPIV-3 such as mikanin sulphate (But et al. 2009), orientin, and vitexin (Li et al. 2002). Chelidimerine, bulbocapnine, norsanguinarine, sanguinarine, protopine, fumariline, bicuculline, adlumidine and palmatine are active (Orhana et al. 2007). Thymol is a strong inhibitor (1.6 µg/mL) whereas the cassane diterpenes caesalmin B (77), bonducellpin D (78), 1α,5α,6α,7β,14β-pentahydroxyvouacapane, 1α,5α-dihydroxy-14β-methoxy-6α,7β-diacetoxyvouacapane (79) as well as friedelin (80) are very strongly active (Jiang et al. 2002; Table 1).

### Respiratory syncytial virus (RSV)

RSV is responsible for cold in adults but cause life-threatening pneumonia in young infants globally. The virion binds to heparan sulphate on the surface of host cell, enters the cell and after capsid disintegration the single-stranded (-)-RNA is released in the cytoplasm where viral RNA-dependent RNA polymerase transcribes it into (+)-RNA (Griffiths et al. 2017).

#### Phenolics

The diarylpropane broussonin A (**81**) from the rhizomes of *Anemarrhena asphodeloides* Bunge (Asparagaceae) (Bae et al. 2007) repressed RSV with the $IC_{50}$ of 0.6 µM whereas cleistocaltone A and B (Song et al. 2019) and grevilloside G have milder potencies (Wang et al. 2013). Perusal of literature indicates that RSV is particularly sensitive to hydroxycinnamic acid derivatives such as 3,5-di-*O*-caffeoylquinic acid (**82**) ($IC_{50}$ 0.6 µg/mL), 1α,2β-*O*-dicaffeoylcyclopentan-3β-ol (Geng et al. 2011), 3,5-di-caffeoyl quinic acid ($IC_{50}$ 2.2 µg/mL; S.I. >90.9) and 3,4-di-caffeoyl quinic acid ($IC_{50}$ 1.8 µg/mL; S.I. >111.1) (Chen et al. 2019). Flavones are in general moderate RSV suppressors such as wogonin, oroxylin, baicalein, scutellarein, baicalin (**93**) (Ma et al. 2002), nobiletin and tangeretin (Xu et al. 2014). Tangeretin given intragastrically at a dose of 100 mg/kg/d to mice for 3 d before intranasal infection with RSV decreased virus count in lung tissues from 3.3 to 3.0 PFU/g $\log^{10}$ (Xu et al. 2015). Another flavonoid acting *in vivo* is the *C*-glycosyl flavonol isoorientin which is *in vitro* is a strong inhibitor of RSV ($IC_{50}$ 2.4 µg/mL). Other phenolic with strong anti-RSV potencies are the neolignans nyasol (**83**) and 4′-*O*-methylnyasol (**84**) from *A. asphodeloides* ($IC_{50}$ 0.8 and 0.3 μM) (Bae et al. 2007) and euparin (**5**) (Wang, Qin, et al. 2016; Wang, Wang, et al. 2016). Mangiferin (Zhang, Wu, et al. 2015; Zhang, Zhang, et al. 2015), piceatannol-3′-*O*-β-D-glucopyranoside (Chen et al. 2019), emodin (**7**) (Liu et al. 2015) have modest potencies.

### Terpenes

*Barleria prionitis* L. (Acanthaceae) yields the iridoid glycoside 6-*O*-*trans*-*p*-coumaroyl-8-*O*-acetylshanzhiside methyl ester ($EC_{50}$ 2.4 μg/mL; S.I. 17) (Chen et al. 1998). Diterpenes are moderate RSV suppressors such as ajacisine E (Yang et al. 2017) and forsypensins A-E (Xiang et al. 2020). The *seco*-dammarane triterpene dammarenolic acid (**85**) from *Aglaia ignea* Valeton ex K. Heyne (Meliaceae) is very strongly active ($IC_{50}$ 0.1 μg/mL: S.I. 29.2) (Esimone et al. 2008). Meliaceous triterpenes are in general strong inhibitors of RSV *in vitro* such as niloticin, piscidol A (Esimone et al. 2008), aglaiol, 24,25-epoxy-dammar-20-ene-3-one and 24,25-dihydroxy-5α-dammar-20-ene-3-one (Esimone et al. 2008). Other instances are 4α,14-dimethyl-9,19-cyclocholestan-3β,24α, 25-triol, 24,25-epoxy-cycloartan-3-ol, lupeol, lupeone (Esimone et al. 2008), dysoxylin A ($EC_{50}$ 2 μg/mL), B ($EC_{50}$ 4 μg/mL) and D ($EC_{50}$ 1 μg/mL) (**86**). As for saponins, timosaponin A-III from *A. asphodeloides* is strongly active ($IC_{50}$ 1 μM) (Youn et al. 2011).

### Vesicular stomatitis virus (VSV)

Horses infected with vesicular stomatitis virus (VSV) develop fever, oral vesicles, anorexia and dehydration while in human the virus causes influenza-like symptoms. No treatment for VSV exists. The viral surface G protein binds to host cell LDL-receptor and the single-stranded (-)-RNA is used as a substrate for the synthesis of (+)-RNA by RNA polymerase (Finkelshtein et al. 2013). In general, secondary metabolites of Angiosperms are moderately active towards VSV *in vitro* such as curcumin (Mounce et al. 2017), baicalein (Zhang et al. 2019), methylgallate (Kane et al. 1988), 1-cinnamoyl-3,11-dihydroxymeliacarpin (Alché et al. 2003), simalikalactone D (Apers et al. 2002), ursanes (Aquino et al. 1995) and glycyrrhizin. Flavonol glycosides, such as cirsitakaoside from *Premna szemaoensis* C. P″ei (Verbenaceae) are active against VSV *in vivo*. Boragineous pyrrolizidine alkaloids, such as heliotrine and 7-angeloyl heliotrine are strongly active (Singh et al. 2002).

### Lassa virus (LASV)

LASV is transmitted from rats and accounts for fatal haemorrhages (Reuben et al. 2020). The amide alkaloid capsaicin from *Capsicum annum* L. (Solanaceae) moderately repressed this virus (Tang, Zhang, et al. 2020).

Sandfly fever Sicilian virus (SFSV), Punta toro virus (PTV) and Rift valley fever virus (RFV)

Amaryllidaceae alkaloids are very strong inhibitors of SFSV, PTV and RFV *in vitro*. SFVS was suppressed by pretazettine (**27**) ($IC_{50}$ 0.8 μg/mL) and lycoricidine (**24**) ($IC_{50}$ 0.005 μg/mL) (Gabrielsen et al. 1992). Narciclasine (**26**), lycoricidine (**24**), pseudolycorine (**23**), and pretazettine (**27**) hindered PTV with $IC_{50}$ values of 0.007, 0.005, 0.6 and 0.6 μg/mL, respectively (Gabrielsen et al. 1992). Pseudolycorine (**23**), pretazettine (**27**), lycoricidine (**24**) and pancrastitatin (**11**) restrained RFV the with the $IC_{50}$ values of 0.6, 2.9, 0.1 and 0.1 μg/mL, respectively (Gabrielsen et al. 1992).

*Influenza virus (IV).* Influenza virus (IV) is zoonotic from aquatic birds and evokes fever, cough and shortness of breath that if not treated in unvaccinated elderly patients may eventually lead to fatal pneumonia. The viral surface haemagglutinin and neuraminidase glycoproteins binds to the host cell sialic acid and after internalisation and disintegration of viral capsid, single-stranded (-)-RNA is translated by viral RNA-dependent RNA polymerase into a (+)-RNA used for the synthesis of protein by host ribosomes (Samji 2009). Perusal of literature indicates that very strong IV inhibitors are mainly phenolics (Table 1).

### Simple phenolics

Simple phenols are mainly strong inhibitors of IV *in vitro* such as gallic acid (**1**) (You et al. 2018), hydroxytyrosol (**36**) (Yamada et al. 2009) and ellagic acid (Chang et al. 2016), the latter active *in vivo* (Park et al. 2013). Protocatechuic acid given at the dose of 20 mg/kg twice daily by oral gavages at 12 h intervals for 7 d protected mice against IV (Ou et al. 2014). Other phenolics inhibiting IV are 4,4′, 7,7′-tetrahydroxy-2,2′, 8,8′-tetramethoxy-1,1′-di-phenanthrene (Langeder et al. 2020) and methyl brevifolincarboxylate (Chen, Yang, Huang, et al. 2020; Chen, Yang, Zhai, et al. 2020).

### Hydroxycinnamic acid derivatives

Apart from 1′-acetoxychavicol ($IC_{50}$ 2 μM) (Watanabe et al. 2011), hydroxycinnamic acid derivatives are mostly moderate repressors of IV such as rosmarinic acid methyl ester I (Bang et al. 2016), and chlorogenic acid (Din et al. 2017). Forsythoside A C is active *in vivo* (Law et al. 2017) as well as cinnamic acid (Hayashi et al. 2007).

### Coumarins

10′*R*-Acetoxy-11′-hydroxyumbelliprenin (**88**), methyl galbanate (**89**), farnesiferol C (**90**) and *epi*-conferdione (**87**) from *Ferula assa-foetida* L. (Apiaceae) very strongly inhibited H1N1 with the $IC_{50}$ values of 0.9, 0.2, 0.2 and 0.3 μg/mL, respectively (Lee et al. 2009). Other anti-IV are scopoletin (Yang et al. 2018), spirotriscoumarin A (Tang et al. 2016) as well as 2(3H)-benzoxazolinone (Gu et al. 2015).

### Flavans

These are mainly moderately to strongly active against IV such as (+)-catechin (You et al. 2018; +)-catechin gallate (Chang et al. 2016), and epigallocatechin-3-*O*-gallate (Derksen et al. 2014).

### Flavones

Quercetin (**91**) reduced IV (H5N1) plaque formation by 68% at 1 ng/mL (Ibrahim et al. 2013; Tian et al. 2011). Myricetin (Pantev et al. 2006), kaempferol, apigenin (Pantev et al. 2006), isoquercetin (Kim et al. 2010), wogonin, chrysin (Kim et al. 2021), hispidulin (Bang et al. 2016), as well as tricin (Yazawa et al. 2011) are strongly active *in vitro*, as well as baicalein (Chen et al. 2011), oroxylin, diplacone and dihydroquercetin. Flavonol glycosides are often very strong repressors of IV such as rutin (**92**) (73.2%

H5N1 reduction at 1 ng/mL) (Ibrahim et al. 2013), baicalin (**93**) ($IC_{50}$ 0.7 μg/mL; S.I. 22.9) (Chen et al. 2011). Of lesser potencies are tamarixetin 3-robinobioside (Nguyen et al. 2016), kaempferol-3-*O*-[2″,6″-di-*O*-*Z*-*p*-coumaroyl]-β-D-glucopyranoside (Kim et al. 2019), 2″-*O*-(2‴- methylbutyryl)-isoswertisin (Cai et al. 2006), 2″,4″- myricetin-3″,5″-dimethylether 3-*O*-β-D-galactopyranoside (H1N1 A/PR/8/34), quercetin 3-*O*-β-D-galactopyranoside and quercetin-3-*O*-galactoside (Pantev et al. 2006). Quercetin-3-*O*-β-D-glucuronide from *Persicaria perfoliata* H. Gross. (Polygonaceae) and given orally at a dose of 6 mg/kg for 4 d protected mice against IV-induced lung edoema by 27.9% (Fan et al. 2011). Another example of flavonol active orally is kaempferol (15 mg/kg/d) in mice infected by H9N2 (Zhang, Ai, et al. 2017; Zhang, Rumschlag-Booms, et al. 2017) and agathisflavone (de Freitas et al. 2020). Examples of isoflavonoids, active *in vivo* are biochanin A (Sithisarn et al. 2013) and puerarin which at the dose of 200 mg/kg/d intraperitoneally for 5 d protected mice by 70% against H1N1.

### Other flavonoids

Chalcones are moderate IV inhibitors such as 2,4′,6′-trihydroxy-3′-prenylchalcone (Meyer et al. 1997), 2′,4′-dihydroxy-6′-methoxy-3′,5′-dimethylchalcone and (*E*)-4,2′,4′-trihydroxy-6′-methoxy-3′,5′-dimethylchalcone (Dao et al. 2010). Sappanone A (**94**) from *Caesalpinia sappan* L. (Fabaceae) are strongly active.

### Lignans

Rhinacanthins E (**11**) and F (**12**) (Kernan et al. 1997 +-pinoresinol 4-*O*-[6″-*O*-vanilloyl]-*β*-D -glucopyranoside (A/PR/8/34 (H1N1) (Parhira et al. 2014 +-pinoresinol-*O*-β-D-glucopyranoside (A/PR/8/34 (H1N1) (Li et al. 2019) are moderate inhibitors.

### Stilbenes

Resveratrol (Kim et al. 2010) and ε-viniferin have moderate activities against IV (Nguyen et al. 2011).

### Benzoquinones

The long-alkylated benzoquinone embelin (**95**) from *Embelia ribes* Burm.f (Myrsinaceae) inhibited IV an $IC_{50}$ of 0.3 μM (Hossan et al. 2018).

### Anthraquinones

The naphthodianthrone hypericin (**96**) in *Homalocladium platycladum* (F.J. Muell.) L.H. Bailey (Polygonaceae) repressed IV (H3N2) with an $IC_{50}$ as low as 2.1 ng/mL (Yasuda et al. 2010). Other anthraquinones with moderate potencies are aloe-emodin (**2**) (Li et al. 2014) and 1,3-dihydroxy-5-methoxy-2,6-bismethoxymethyl-9,10-anthraquinone (Wang, Qin, et al. 2016; Wang, Wang, et al. 2016).

### Tannins

1,3,4,6-Tetra-*O*-galloyl-β-D-glucopyranoside (**97**) from *Euphorbia hirta* L. (Phyllanthaceae) yielded the $EC_{50}$ 0.3 μM with IV (Chang et al. 2016). Other anti-IV gallotannins are penta-*O*-galloyl-glucose (Abdel-Mageed et al. 2014) and pentagalloyl-glucose (Derksen et al. 2014). Likewise, ellagitannins are moderate potencies as seen in isocorilagin (Chen, Yang, Huang, et al. 2020; Chen, Yang, Zhai, et al. 2020), corilagin and chebulagic acid (Dao et al. 2018). Weaker activities are obtained with condensed tannins such as procyanidin B2-di-gallate (Derksen et al. 2014). Theaflavin-3,3′-digallate (**98**) from *C. sinensis* very strongly inhibited IV ($IC_{50}$ 0.7 μg/mL).

### Alkaloids

Berberine (**99**) yielded the $IC_{50}$ value of 0.01 μM and given intraperitoneally to mice, at a dose of 5 mg/kg/d for 7 d decreased influenza related mortality rate from 90% to 55% in rodent (Shao et al. 2020). Other isoquinolines of interest are lycorine (**3**) ($IC_{90}$ 0.5 μM; S.I. >45) as well as (-)-thalimonine (**100**) from *Thalictrum simplex* L. (Ranunculaceae) ($EC_{50}$ 0.1 μM; S.I. 640) (Serkedjieva and Velcheva 2003). *Mitragyna speciosa* (Korth.) Havil. (Rubiaceae) yields hirsutine (**101**) ($IC_{50}$ 0.4 μg/mL; S.I. 58.8) (Tietze and Zhou 1999). The quinazoline alkaloid camptothecin is active against IV (Kelly et al. 1974) as well as melicopteline C (Lee et al. 2021), dendrobine ($IC_{50}$ 2.1 μg/mL) (Li et al. 2017) and homonojirimycin (Zhang, Liu, et al. 2013; Zhang, Li, et al. 2013).

### Terpenes

Geniposide given intraperitoneally at the dose of 20 mg/kg/d increased the survival rate of mice experimentally infected with the pandemic IV (A/Jiangsu/1/2009 (H1N1) (Zhang et al. 2017; Zhang, Rumschlag-Booms, et al. 2017). As for sesquiterpenes, reynoudiol (**102**) from *Polygonum cuspidatum* Siebold and Zucc. (Polygonaceae) strongly inhibited IV ($IC_{50}$ 0.2 μM; S.I. >100) (Nhiem et al. 2014). Other strong inhibitors are phyllaemblicin B ($IC_{50}$ 2.6 μg/mL) and atractylon, the latter active orally in mice (Chen et al. 2016). Of weaker potencies are gossypol (Dorsett et al. 1975) and β-santalol (Paulpandi et al. 2012). Within the diterpenes, andrographolide as well as forsypensins A–E (Xiang et al. 2020) are weakly active. The limonoid thaixylomolin I, K and M (Li et al. 2015) and the lupane triterpene paracaseolin A (Gong et al. 2017) displayed some levels if activity. Saponins are active *in vivo*, such as gymnemic acid A (Sinsheimer et al. 1968) or polyphylla saponin I from *Paris chinensis* Franch. (Melanthiaceae) (Pu et al. 2015). Glycyrrhizin has weak potencies (Harada 2005).

## The distribution of anti-RNA virus natural products among the various clades of angiosperms in Asia and the Pacific

Regarding the distribution of anti-RNA natural products (Tables 2 and 3), the following observations can be made: (i) All Clades except the Protomagnoliids yield inhibitors of both (+) and (−)-RNA viruses; (ii) The strongest inhibitors of RNA viruses are mainly found in Basal Angiosperms; (iii) Some Clades yield specific antiviral natural products, such as dibenzocyclooctadiene lignans in the Protomagnoliids, oligostilbenes in the Rosids or quinolizidines and quinolizidines and isoflavones in the Fabids, naphthylisoquinoline alkaloids in the Malvids, monoterpene indole alkaloids and quinazoline alkaloids in the Lamiids; (iv) Anti-RNA virus phenolics, flavonoids and triterpenes are in general widespread; (v) Basal Angiosperms use mainly sesquiterpenes, lignans and isoquinolines as phytoalexins; (vi) Core

**Table 2.** Distribution of secondary metabolites with activity against (+)-RNA viruses.

| Clade | Secondary metabolites | Viruses |
|---|---|---|
| Protomagnoliids | | |
| | Dibenzocyclooctadiene lignans | HIV |
| | Tetrahydrofuran lignans | CV and HIV |
| | Lindenane sesquiterpenes | HCV and HIV |
| Magnoliids | | |
| | Dinenzylbutyrolactone lignans | HIV |
| | Condensed tannins | SARS-CoV |
| | Bisbenzylisoquinoline alkaloids | HcoV |
| | Phenanthroindolizidine alkaloids | HIV |
| | Kaurane diterpenes | HIV |
| | Lanostane triterpenes | HIV |
| Monocots | | |
| | Diarylheptanoids | CHIKV and ZIKV |
| | Hydroxycinnamic acid derivatives | DV and HIV |
| | Flavonols | HIV |
| | Chalcones | SARS-CoV |
| | Stilbenes | HIV |
| | Neolignans | DV |
| | Naphthoquinones | HIV |
| | Anthraquinones | HIV, JEV and PV |
| | Amaryllidaceae alkaloids | DV, EV-71, JEV, HIV and PV<br>SFV, WNV, YFV, SARS- CoV and ZIKV |
| | Spirostane saponins | HCV |
| Eudicots | | |
| | Flavonol glycosides | HIV |
| | Benzylisoquinoline alkaloids | HIV |
| | Bisbenzylisoquinoline alkaloids | HIV and JEV |
| | Protoberberine alkaloids | CHIKV, DV, HIV, WNV and ZIKV |
| | Cycloartane saponins | HCV |
| Core Eudicots | | |
| | Simple phenolics | DV, EV-71, HCV and HIV |
| | Phenolic glycosides | CV |
| | Flavonols | SARS-CoV |
| | Gallotannins | HCV |
| Rosids | | |
| | Stilbenes | CHIKV |
| | Oligostilbenes | HCV |
| Fabids | | |
| | Simple phenolics | SARS-CoV |
| | Phenolic glycosides | DV |
| | Phloroglucinols | HIV |
| | Hydroxycinnamic acid derivatives | HIV |
| | Coumarins | HIV, SARS-CoV |
| | Flavonol glycosides | HRV |
| | Isoflavonols | HIV |
| | Bisflavonols | SARS-CoV |
| | Chalcones | SARS-CoV |
| | Chromones | HIV |
| | Oligostilbenes | HIV |
| | Xanthones | HcoV and HIV |
| | Gallotannins | HIV |
| | Ellagitannins | HIV |
| | Indole alkaloids | HIV |
| | Quinolizidine alkaloids | EV-71 and HIV |
| | Quinazoline alkalaloids | HcoV |
| | Sesquiterpene pyridine alkaloids | HIV |
| | Norbisabolane sesquiterpene glycosides | EV-71 |
| | Abietane diterpenes | CHIKV and SINV |
| | Tigliane diterpenes | CHIKV and SINV |
| | Daphnane diterpenes | CHIKV |
| | Lupane triterpenes | DV |
| | Ursane triterpenes | DV |
| | Friedelane triterpenes | HcoV |
| | Cycloartane triterpenes | HRV |
| | Oleanane tsaponins | DV, HIV and SARS-CoV<br>WNV and ZIKV |
| Malvids | | |
| | Simple phenolics | SARS-CoV |
| | Flavanols | SARS-CoV and MAYV |
| | Flavanonols | SARS-CoV, JEV, YFV, WNV and ZIKV |
| | Flavonols | HcoV, SARS-CoV, JEV, ZIKV and HIV |
| | Isoflavonols | HRV |
| | Anthraquinones | PV |

(*continued*)

**Table 2.** Continued.

| Clade | Secondary metabolites | Viruses |
|---|---|---|
| | Lignans | CHIKV, HcoV, HCV, HRV, MERS-CoV and ZIKV |
| | Xanthones | HIV |
| | Condensed tannins | SARS-CoV |
| | Ellagitannins | HCV |
| | Gallotannins | SARS-CoV |
| | Acridone alkaloids | HCV |
| | Benzophenanthridine alkaloids | HIV |
| | Indole alkaloids | HIV |
| | Quinoline alkaloids | HIV |
| | Quinolinone alkaloids | HCV and HIV |
| | Cadinane sesquiterpenes | HIV |
| | Guaiane sesquiterpenes | HIV |
| | Tigliane diterpenes | HIV |
| | Dammarane triterpenes | HIV |
| | Limonoids | DV and HIV |
| | Tirucallane triterpenes | DV and YFV |
| | Quassinoids | SFV |
| | Dammarane saponins | MEV |
| Asterids | | |
| | Condensed tannins | RTV and SARS-CoV |
| | Iridoid glycosides | CV, HCV and SARS-CoV |
| | Cadinane sesquiterpenes | CV |
| | Oleanane triterpenes | PEDV |
| Lamiids | | |
| | Hydroxycinnamic acid derivatives | HIV and SFV |
| | Flavanols | HcoV and SARS-CoV |
| | Flavonols | CHIKV, CV, DV, EV-71, HCV and SARS-CoV |
| | Arylnaphthalene lignans | HIV and ZIKV |
| | Dibenzylbutane lignans | CV |
| | Dibenzylbutyrolactone lignans | HCV |
| | Naphthoquinones | JEV and CV |
| | Xanthones | HIV |
| | Monoterpene indole alkaloids | DV and EV-71 |
| | Steroidal alkaloids | ZIKV |
| | Iridoid glycosides | SARS-CoV |
| | Cembrane sesquiterpenes | HIV |
| | Labdane diterpenes | HIV and SARS-CoV |
| | Ursane triterpenes | CV and EV-71 |
| | Ergostane steroids | SARS-CoV |
| | Cardenolides | CHIKV and HIV<br>MERS and SARS<br>CoV and ZIKV |
| Campanulids | | |
| | Coumarins | HCV |
| | Flavonols | HRV and PV |
| | Benzofuran lignans | JEV |
| | Sesquiterpene lactones | HCV and SARS-CoV |
| | Dammarane saponins | EV-71 |
| | Oleanane saponins | HCV |
| | Ursane saponins | HIV |

Angiosperm use mainly phenolics as phytoalexins; (vii) Upper Angiosperm use mainly hydroxycinnamic acid derivatives, iridoid glycosides, indole alkaloids, diterpenes, triterpene saponins and acetylenic fatty acids as phytoalexins; (viii) Very strong anti-RNA natural products occur mostly in medicinal plants used for microbial infection (Table 4).

## The strongest anti-RNA virus natural products from the flowering plants of Asian and the Pacific

Compared with antibacterial or antifungal principles from plants, no accepted classification criteria for *in vitro* strength of antiviral activity seem to be available. We therefore suggest for a compound: (i) $IC_{50}$ value below or equal to 1 µg/mL = very strongly active, (ii) for an $IC_{50}$ value above 1 and equal to or below 20 µg/mL = strongly active, (iii) for an $IC_{50}$ above 20 and below or equal to 100 µg/mL = moderately active, (iv) for an $IC_{50}$ above 100 and below or equal to 500 µg/mL = weakly active, (v) for an $IC_{50}$ ranging from above 500 to below or equal to 2500 µg/mL = very weakly active and, inactive, an $IC_{50}$ values above 2500 µg/mL. Accordingly, out of about the 350 molecules characterised, 102 (43 phenolics, 31 alkaloids and 28 terpenes) are very strongly active against at least one type of RNA virus (Table 1, Figure 1). The lowest $IC_{50}$ values were achieved by oleandrin (**74**) (Plante et al. 2021), silvestrol (**8**) (Müller et al. 2018; Henss et al. 2018), pseudolycorine (**23**) and lycoricidine (**24**), pancratistatin (**25**), narciclasine (**26**), stelleracin A (**67**), B (**68**), and C (**69**), quercetin (**91**) (Ibrahim et al. 2013), rutin (**92**), (Ibrahim et al. 2013) and hypericin (**96**) (Yasuda et al. 2010).

## Spectrum of activity

As for the spectrum of activity, the following observation are made: (i) Tannins and saponins are not very strongly active

Table 3. Distribution of secondary metabolites with activity against (−)-RNA viruses.

| Clade | Secondary metabolites | Viruses |
|---|---|---|
| Magnoliids | | |
| | Amide alkaloids | LASV |
| Monocots | | |
| | Diarylheptanoids | VSV |
| | Simple phenolics | IAV and RSV |
| | Hydroxycinnamic acid derivatives | IAV and RSV |
| | Flavonols | IAV |
| | Flavonol glycosides | RSV |
| | Anthraquinones | IAV |
| | Neolignans | RSV |
| | Amaryllidaceae alkaloids | IAV, RFV, PTV and SFVS |
| | Dendrobine alkaloids | IAV |
| | Piperidine alkaloids | IAV |
| | Spirostane saponins | IAV and RSV |
| Eudicots | | |
| | Pavine alkaloids | IAV |
| | Protoberberine alkaloids | IAV and HIPV-3 |
| | Lycanotinine diterpenes | RSV |
| | Cycloartane triterpenes | RSV |
| Core Eudicots | | |
| | Simple phenolics | EBOV, IAV and VSV |
| | Phenolic glycosides | NDV |
| Fabids | | |
| | Simple phenolics | IAV and RSV |
| | Flavonol glycosides | IAV |
| | Isoflavonols | IAV |
| | Homoisoflavonoids | IAV |
| | Gallotannins | IAV |
| | Norbisabolane sesquiterpene glycosides | IAV |
| | Cassane diterpenes | HIPV-3 |
| | Friedelane triterpenes | HIPV-3 |
| | Oleanane triterpenes | NDV and VSV |
| Malvids | | |
| | Simple phenolics | IAV and RS |
| | Coumarins | IAV |
| | Flavonols | RSV |
| | Flavonol glycosides | IAV |
| | Chalcones | IAV |
| | Chromones | HIV |
| | Lignans | EBOV |
| | Stilbene glycoside | HIPV-3 |
| | Anthraquinones | IAV and HIPV-3 |
| | Naphthoquinones | MEV |
| | Xanthones | HIPV-3 |
| | Ellagitannin | IAV |
| | Cyclopeptide alkaloids | IAV |
| | Sesquiterpenes | IAV |
| | Cholestane steroids | RSV |
| | Cycloartane triterpenes | RSV |
| | Dammarane triterpenes | RSV |
| | Limonoids | RSV and VSV |
| | Lupane triterpenes | IAV and RSV |
| | Quassinoids | VSV |
| | Cycloartane saponins | MEV |
| Asterids | | |
| | Simple phenolics | IAV |
| | Flavanols | IVA |
| | Long-chain benzoquinones | IVA |
| | Condensed tannins | IVA |
| | Quinazoline alkaloids | IVA |
| Lamiids | | |
| | Hydroxycinnamic acid derivatives | IAV and RSV |
| | Flavonols | RSV, HIPV-3 and IVA |
| | Flavonol glycosides | VSV |
| | Dibenzylbutane lignans | IVA |
| | Furofuran lignans | IVA |
| | Monoterpene indole alkaloids | IAV |
| | Monoterpenes | HIPV-3 |
| | Iridoid glycosides | HIPV-3 |
| | Labdane diterpenes | IAV and RSV |
| | Oleanane saponins | IAV |
| Campanulids | | |
| | Hydroxycinnamic acid derivatives | IAV |
| | Coumarins | IAV |
| | Flavonols | RSV |

Table 4. Medicinal plants of Asia and the Pacific yielding anti-RNA principles.

| Clade | Family | Genus, species | Local name | Use | Country |
|---|---|---|---|---|---|
| Protomagnoliids | Chloranthaceae | *Chloranthus japonicus* Siebold | Yin xian cao | Cough | China |
| | Saururaceae | *Houttuynia cordata* Thunb. | Dokudami | Infection | Japan |
| | | *Saururus chinensis* (Lour.) Baill. | San bai cao | Boils | China |
| | Schisandraceae | *Kadsura longipedunculata* Finet & Gagnep. | | | |
| | | *Schisandra rubriflora* (Franch.) Rehder & E.H. Wilson | | | |
| Magnoliids | | | | | |
| | Annonaceae | *Annona squamosa* L. | Ates | Fever | the Philippines |
| | | *Polyalthia suberosa* (Roxb.) Thwaites | Dudduga chettu | Puerperal fever | India |
| | Lauraceae | *Cryptocarya chinensis* (Hance) Hemsl. | | | |
| | | *Cinnamomum cassia* (L.) J. Presl | Kayu manis | Cough | Malaysia |
| | | *Litsea verticillata* Hance | | | |
| | Magnoliaceae | *Magnolia officinalis* Rehder & E.H. Wilson | Hou po | Cold | China |
| | Piperaceae | *Piper cubeba* L. | Walga-miris | Gonorrhea | Sri Lanka |
| Monocots | Acoraceae | *Acorus calamus* L. | Jerango | Boils | Indonesia |
| | Asparagaceae | *Anemarrhena asphodeloides* Bunge | Zhi mu | Influenza | China |
| | Aspholedaceae | *Aloe vera* (L.) Burm. F. | Ghrita koomari | Dysentery | Bangladesh |
| | Dioscoreaceae | *Dioscorea batatas* Decne. | Shu yu | Abscesses | China |
| | Iridaceae | *Eleutherine americana* (Aubl.) Merr. Ex K. Heyne | | Flu | Thailand |
| | Melanthiaceae; | *Paris chinensis* Franch. | | Cough | Nepal |
| | Xanthorrhoeaceae | *Hemerocallis fulva* (L.) L. | Xuan cao | Fever | China |
| | Zingiberaceae | *Alpinia galanga* (L.) Willd. | Kelawas | Herpes | Indonesia |
| | | *Boesenbergia rotunda* (L.) Manfs. | Kra chai | Dysentery | Thailand |
| Eudicots | | | | | |
| | Menispermaceae | *Cissampelos hirsuta* Buch.-Ham. Ex DC. | Peepra mool | Fever | India |
| | | *Stephania rotunda* Lour. | | | |
| | Nelumboaceae | *Nelumbo nucifera* Gaertn. | Lan | Pneumonia | China |
| | Ranunculaceae | *Cimicifuga foetida* L. | | | |
| | | *Thalictrum simplex* L. | Jian tou tang song cao | Infections | China |
| Core Eudicots | Crassulaceae | *Rhodiola rosea* L. | | | |
| | Lardizabalaceae | *Sargentodoxa cuneata* (Oliv.) Rehder & E.H. Wilson | | | |
| | Saxifragaceae | *Saxifraga melanocentra* Franch. | | | |
| Fabids | Begoniaceae | *Begonia nantoensis* M.J. Lai & N.J. | | | |
| | Celastraceae | *Tripterygium wilfordii* Hook. F. | | | |
| | Clusiaceae | *Calophyllum inophyllum* L. | Ponenyet | Eye infection | Myanmar |
| | | *Garcinia sp.* | | | |
| | | *Mesua ferrea* L. | Nageswar | Leucorrhoea | India |
| | Euphorbiaceae | *Croton sp.* | | | |
| | | *Euphorbia hirta* L. | Amumpatchai | Cold | India |
| | | Excoecaria acerifolia Didr. | | | |
| | | *Excoecaria agallocha* L. | Sinu gaga | Leprosy | Fiji |
| | | *Euphorbia neriifolia* L. | Snuhi | Cough | India |
| | | *Mallotus philippensis* (Lam.) Müll. Arg. | Kampillaka | Cough | India |
| | | *Trigonostemon thyrsoideus* Stapf | | | |
| | Hypericaceae | *Hypericum japonicum* Thunb. | Di er cao | Hepatitis | China |
| | Juglandaceae | *Juglans mandshurica* Maxim. | Hu tao qiu | Skin infection | China |
| | Fabaceae | *Adenanthera pavonina* L. | Raktochandan | Eye infection | Bangladesh |
| | | *Astragalus mongholicus* Bunge | Huangqi | Dysentery | China |
| | | Caragana rosea Turcz. Ex Maxim. | Hong hua jin ji er | | China |
| | | *Caesalpinia sappan* L. | | | |
| | | Indigofera tinctoria L. | Nilayamari | Bronchitis | India |
| | | *Sophora flavescens* Aiton | | | |
| | | *Sophora japonica* L. | | | |
| | Fagaceae | *Castanea crenata* Siebold & Zucc. | Ri ben li | Tuberculosis | China |
| | Flacourtiaceae | *Flacourtia sp.* | | | |
| | Malpighiaceae | *Galphimia glauca* Cav. | | Wounds | Bangladesh |
| | Phyllanthaceae | *Phyllanthus emblica* L. | Kada mola | Jaundice | Bangladesh |
| | | *Phyllanthus niruri* L. | Dukong anak | Jaundice | Malaysia |
| Malvids | Amaranthaceae | Alternanthera philoxeroides (Mart.) Griseb. | Xi han lian zi cao | Fever | China |
| | Anacardiaceae | *Rhus javanica* L. | | | |
| | Ancistrocladaceae | *Ancistrocladus sp.* | | | |
| | Caryophyllaceae | *Drymaria cordata* (L.) Willd. Ex Schult. | Peperano | Cough | Nepal |
| | Combretaceae | *Terminalia chebula* Retz. | Abhaya | Bronchitis | India |
| | Dipterocarpaceae | *Dryobalanops aromatica* C.F. Gaertn. | | | |
| | | *Vatica* L. | | | |
| | Droseraceae | *Drosera peltata* Thunb. | Mukhjali | Bronchitis | India |
| | Loranthaceae | *Taxillus sutchuenensis* (Lecomte) Danser | | | |
| | Lythraceae | *Lagerstroemia speciosa* (L.) Pers. | | | |
| | Malvaceae | *Gossypium arboreum* L. | Kaarpaasi | Fever | India |
| | | Melochia odorata L.f. | Ulari | UTI | Papau |
| | | *Morus alba* L. | Toot | Sore throat | India |
| | Meliaceae | *Aglaia ignea* Valeton ex K. Heyne | | | |
| | | *Dysoxylum gaudichaudianum* Miq. | | | |
| | | *Melia azedarach* L. | | | |

(*continued*)

**Table 4.** Continued.

| Clade | Family | Genus, species | Local name | Use | Country |
|---|---|---|---|---|---|
| | | *Swietenia macrophylla* King | | | |
| | | *Swietenia mahagoni* (L.) Jacq. | Ciminukku | Fever | India |
| | Myrsinaceae | *Embelia ribes* Burm.f | Vidanga | Flu | India |
| | Myrtaceae | *Rhodomyrtus tomentosa* (Aiton) Hassk | | | |
| | Olacacaeae | *Onachanostachys amentacea* Mast. | | | |
| | Plumbaginaceae | *Plumbago indica* L. | Rakta-chitraka | Fever | India |
| | Polygonaceae | *Homalocladium platycladum* (F.J. Muell.) L.H. Bailey | Jangkang | Wounds | Indonesia |
| | | *Persicaria perfoliata* H. Gross. | Gang ban gui | Tonsillitis | China |
| | | *Polygonum capitatum* Buch.-Ham. Ex D. Don | Tou hua liao | Fever | China |
| | | *Polygonum cuspidatum* Siebold & Zucc. | | | |
| | | *Rheum australe* D. Don | Revatchini | Bronchitis | India |
| | | *Rheum palmatum* L. | Rivand-i-chini | Jaundice | Iran |
| | Rutaceae | *Aegle marmelos* (L.) Corrêa | Mak phyn | Bronchitis | Myanmar |
| | | *Boenninghausenia albiflora* (Hook.) Rchb. Ex Meisn. | Makhamar | Fever | Nepal |
| | | *Citrus* × *bergamia* Risso & Poit | | | |
| | | *Citrus sinensis* (L.) Osbeck | Naranj | Dysentery | Irak |
| | | *Citrus trifoliata* L. | Zhi | COVID-19 | China |
| | | *Clausena excavata* Burm.f. | Daw-hke | Leprosy | Myanmar |
| | | *Glycosmis montana* Pierre | | | |
| | | *Melicope latifolia* (DC.) T.G. Hartley | | | |
| | | *Ruta angustifolia* (L.) Pers. | Godong minggu | Jaundice | Indonesia |
| | | *Zanthoxyllum ailanthoides* Siebold & Zucc | | | |
| | | *Zanthoxylum piperitum* (L.) DC. | Chopinamu | Cat bites | Korea |
| | Simaroubaceae | *Quassia sp.* | | | |
| | Thymeleaceae | *Stellera chamaejasme* L. | Ri jia aa | Ulcers | China |
| Asterids | Cornaceae | *Alangium chinense* (Lour.) Harms | Ba jiao feng | Fever | China |
| | | *Cornus officinalis* Sieb. & Zucc. | Shan zhu yu | Fever | China |
| | Ebenaceae | *Diospyros sp.* | | | |
| | Theaceae | *Camellia sinensis* (L.) Kuntze | Ca | Eye infection | Japan |
| | | *Camellia japonica* L. | Tsubaki | Skin care | Japan |
| Lamiids | Acanthaceae | *Andrographis paniculata* (Burm. F.) Wall. Ex Nees | Hempedu pahit | COVID-10 | Malaysia |
| | | *Barleria prionitis* L. | Peetjhanti | Cough | Bangladesh |
| | | *Justicia gandarussa* L. | Jogut-mudun | Wounds | Bangladesh |
| | | *Ruellia tuberosa* L. | Toi ting | Ulcers | Thailand |
| | | *Rhinacanthus nasutus* (L.) Kurz. | | Herpes | Thailand |
| | Apocynaceae | | | | |
| | | *Holarrhena pubescens* Wall. Ex G. Don | Dangkyam | Boils | Bangladesh |
| | | *Nerium oleander* L. | Nwei thargi | Ringworm | Myanmar |
| | | *Trachelospermum jasminoides* (Lindl.) Lem. | Luo shi | Gonorrhoea | China |
| | Bignonaceae | *Kigelia pinnata* (Jacq.) DC. | Jhaar phaanuus | Dysentery | India |
| | Boraginaceae | *Lithospermum erythrorhizon* Siebold & Zucc. | | Smallpox | Korea |
| | | *Tournefortia sarmentosa* Lam | Salakapo kapu | Fever | Papua |
| | Gentianaceae | *Gentiana macrophylla* Pallas | Qin jiao | Tuberculosis | China |
| | Lamiaceae | *Hyptis capitata* Jacq. | | Influenza | Taiwan |
| | | *Salvia miltiorrhiza* Bunge | Dan shen | COVID-19 | China |
| | | *Ocimum basilicum* L. | Arjaka | Dysentery | India |
| | | *Scutellaria baicalensis* Georgi | Huang qin | COVID-19 | China |
| | Oleaceae | *Forsythia* sp. | | | |
| | | *Nyctanthes arbor-tristis* L. | Seik-palu | Dysentery | Myanmar |
| | Rubiaceae | Guettarda speciosa L. | Hinma | Wounds | India |
| | | *Mitragyna speciosa* (Korth.) Havil. | | | |
| | Solanaceae | *Capsicum annum* L. | Lodachina | Sore throat | India |
| | Verbanaceae | *Premna szemaoensis* C. P'ei | | | |
| Campanulids | Apiaceae | *Ferula assa-foetida* L. | | | |
| | Araliaceae | *Panax ginseng* C.A Meyer | Renshen | COVID-19 | China |
| | Asteraceae | *Cynara scolymus* L. | Kangar firangi | Jaundice | Turkey |
| | | *Arctium lappa* L. | Niu bang | COVID-19 | China |
| | | *Artemisia vulgaris* L. | | | |
| | | *Eupatorium chinense* L. | | | |
| | | *Inula japonica* Thunb. | Xuan fu hua | Abscesses | China |
| | | Laggera pterodonta (DC.) Sch. Bip. Ex Oliv. | | | |
| | | *Spilanthes paniculata* Wall. Ex DC | | Dysentery | India |
| | Campanulaceae | *Platycodon grandiflorus* (Jacq.) A. DC. | Do ra ji | Cold | Korea |

against non-enveloped viruses suggesting that they tend to act by inhibiting viral anchoring and fusion (non-enveloped viruses do not undergo fusion); (ii) A few natural products very strongly repress non-enveloped (+)-RNA viruses; (iii) None of the alkaloids identified showed strong activity against enveloped, monopartite, linear, single-stranded (-)-RNA viruses; and (iv) silvestrol (**8**), lycorine (**3**) and lycoricidine (**24**) inhibit a broad spectrum of RNA viruses.

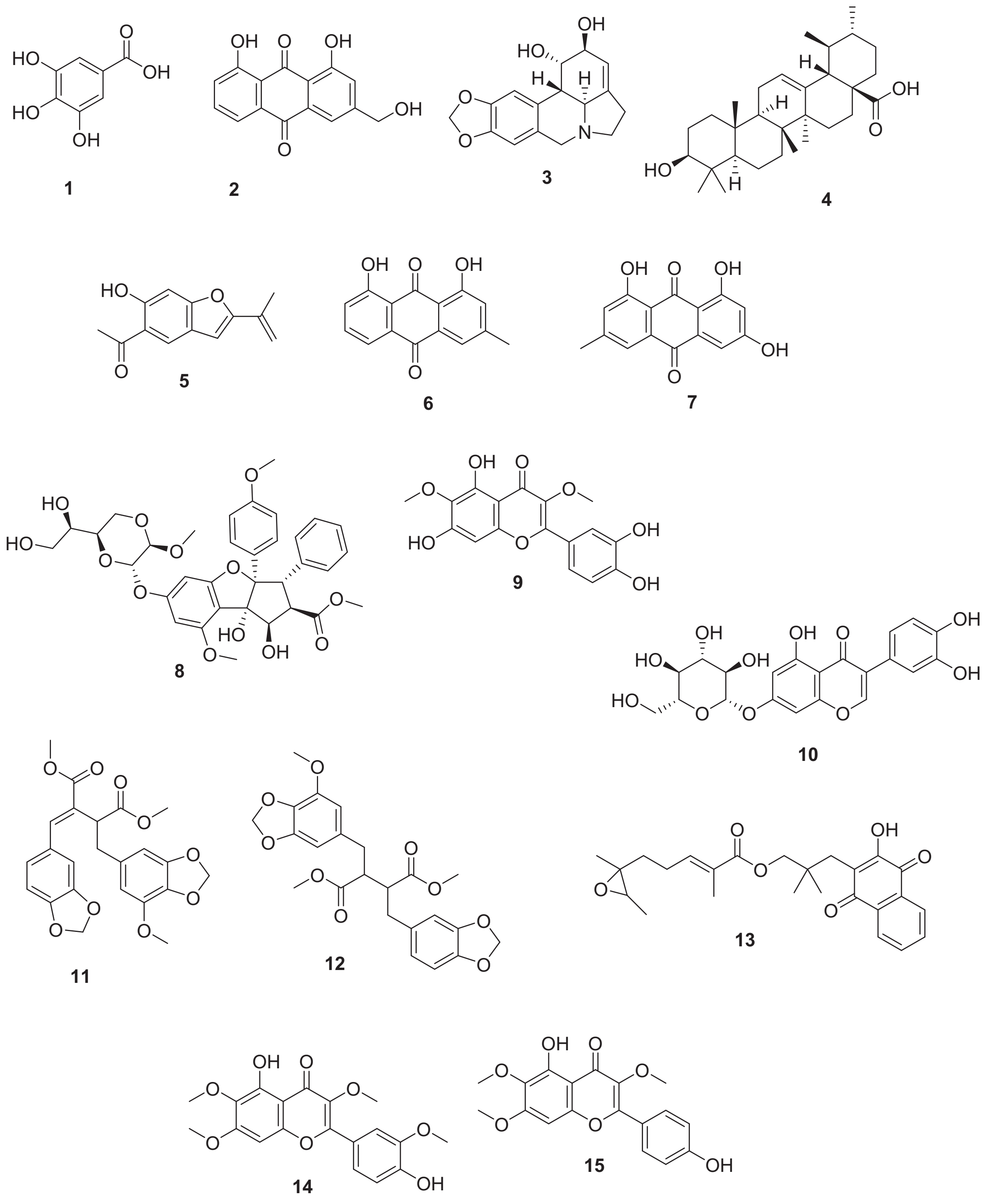

Figure 1. Chemical structures of very strong inhibitors of RNA viruses from the medicinal plants of Asia and the Pacific.

## The influence of molecular mass

The molecular mass of natural products tends to influence their ability to fit in the catalytic pockets of enzymes and to cross biological membranes. Here, we define low molecular mass molecules with a molecular mass below 200 g/mol, medium molecular mass molecules with a molecular mass from 200 to 400 g/mol, and high molecular mass molecules with a molecular mass above 400 g/mol (Table 1). Following this classification, we note that principles with very strong activity have mainly a medium to high molecular mass.

## The influence of solubility: the importance of amphiphilicity

The water solubility of natural products dictates their ability to cross biological membranes. For instance, lipophilic molecules

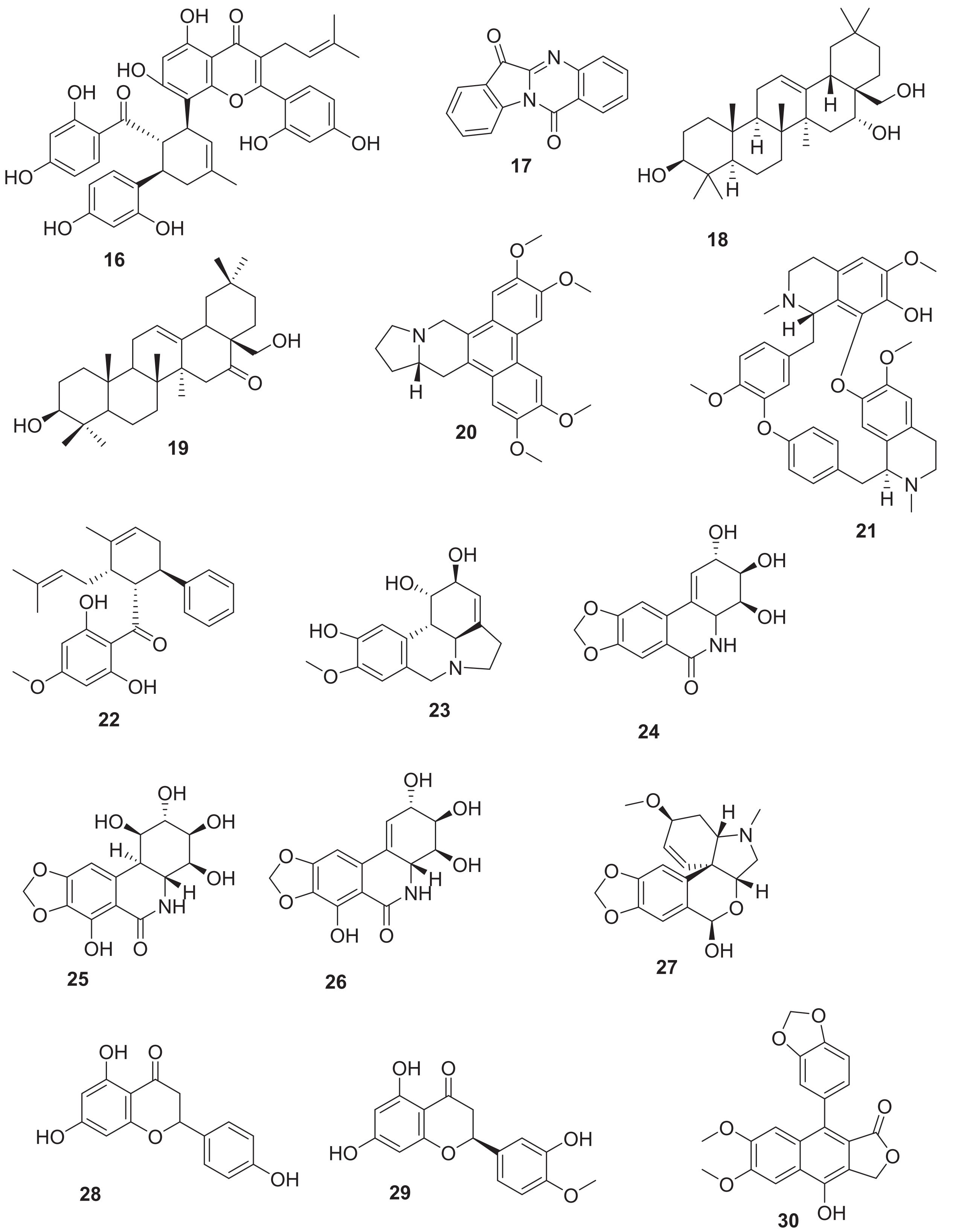

Figure 1. (Continued).

and to a lesser extend amphiphilic molecules can penetrate cells via passive diffusion whereas large hydrophilic molecules require active transports or pinocytosis (Rustad 1961). Log P is equal to the ratio of concentrations of a compound between octanol and water. Hydrophilic compounds (hydrophilic) have low or negative values (about −3) (compounds are mainly found in the

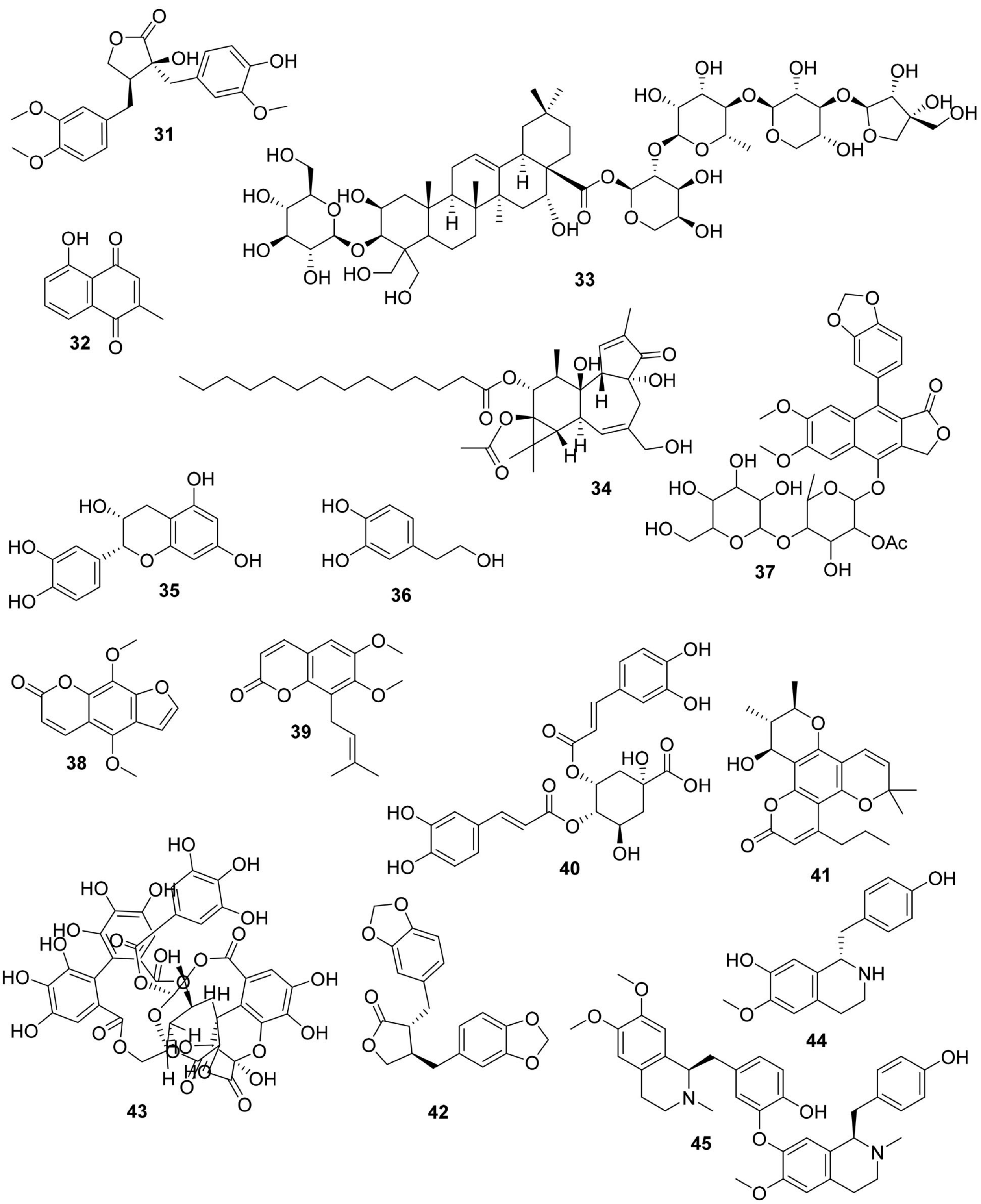

**Figure 1.** (Continued).

water phase). Mid-hydrophilic compounds have Log P is near to 0 (the compound is equally partitioned between the octanol and water layers). Non-hydrophilic (hydrophobic, liposoluble) compounds have a high Log P (up to about 7) (note that lipophilic natural products may tend to remain in and to destabilise the outer membrane of enveloped viruses and/or host cells). However, Log P is only relevant for non-ionizable principles and for ionised substance (such as berberine (**98**) has a Log D is preferable at pH 7.4. Here we define at pH 7.4 hydrophilic compounds for a negative LogD to a value 1, amphiphilic (mid--polar) compounds for a LogD above 1 up to about 4.5, and lipophilic for a LogD above about 5. Note that LogD values given here are predicted values. Accordingly, molecules with very strong activity are mainly hydrophilic or amphiphilic.

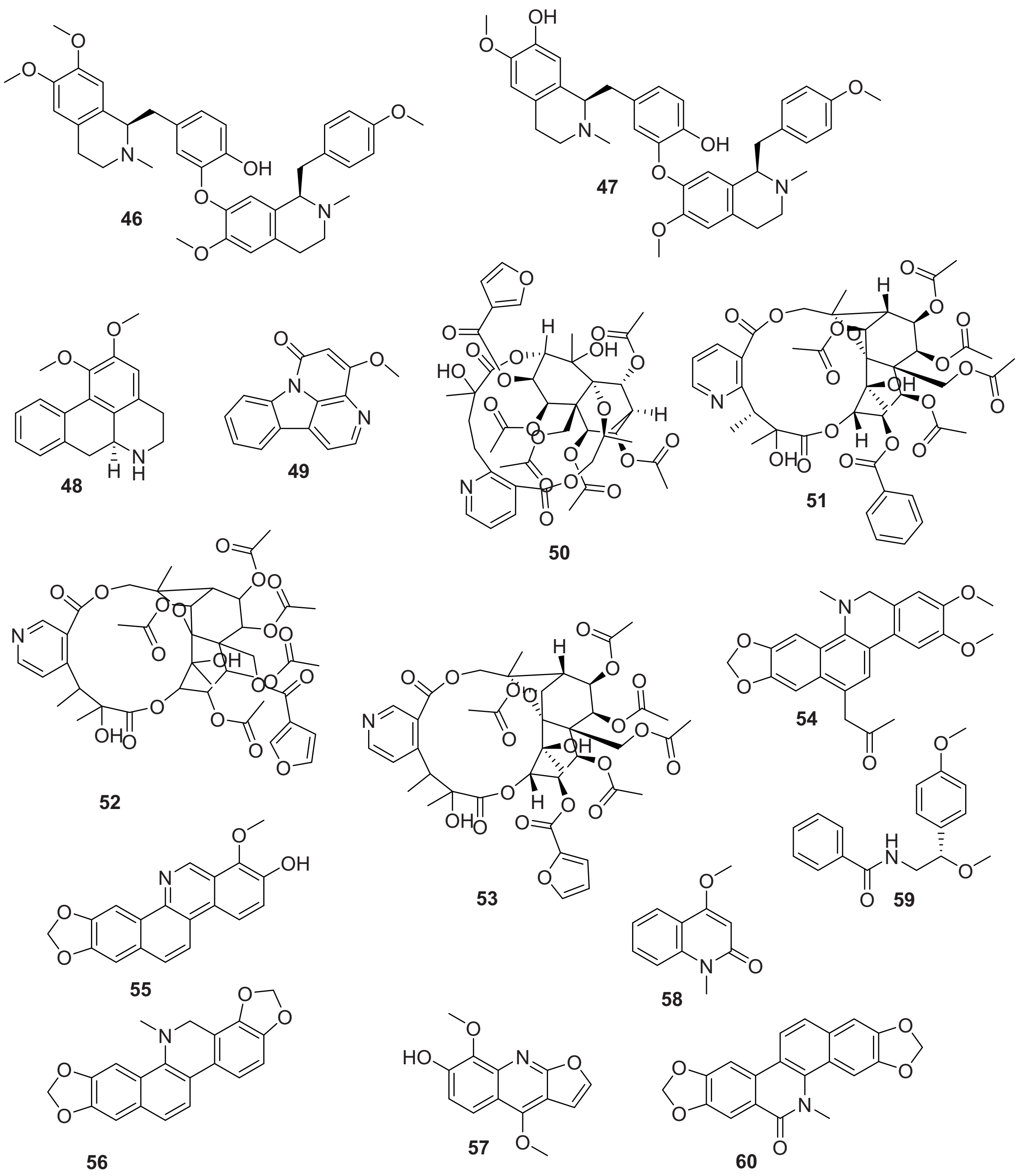

Figure 1. (Continued).

## Mechanism of action: non-specific and/or specific targets

Natural products from the medicinal plants of Asia and the Pacific have, depending on their chemical structure, non-specific and or specific mechanism of annihilation of RNA viruses.

### *Virucidal activity*

Mainly phenolics such as baicalein (Johari et al. 2012), epicatechin (**35**) (Ferraz et al. 2019), hesperitin (**29**) (Castrillo et al. 2015), silvestrol (**8**) (Elgner et al. 2018), gallic acid (**1**) (Hsu et al. 2015), and hydroxytyrosol (**36**) (Yamada et al. 2009). Flavanols evoke viral envelope desquamation, shape distortion, and destruction as well as flavones. Tannins are virucidal such as 1,2,4,6-tetra-*O*-galloyl-β-D-glucose, *ent*-epiafzelechin-(4α→8)-epi-afzelechin (Cheng et al. 2005), pterocarnin A, and proanthocyanidin A-1 (Cheng et al. 2005). Anthraquinones are virucidal as well as gossypol (Polsky et al. 1989), polyphylla saponin I (Pu et al. 2015), and glycyrrhizin, the latter targeting a broad-spectrum of viruses. Non-enveloped viruses resist virucidal natural products. Alkaloids do not seem to be virucidal. Gallic acid (**1**)

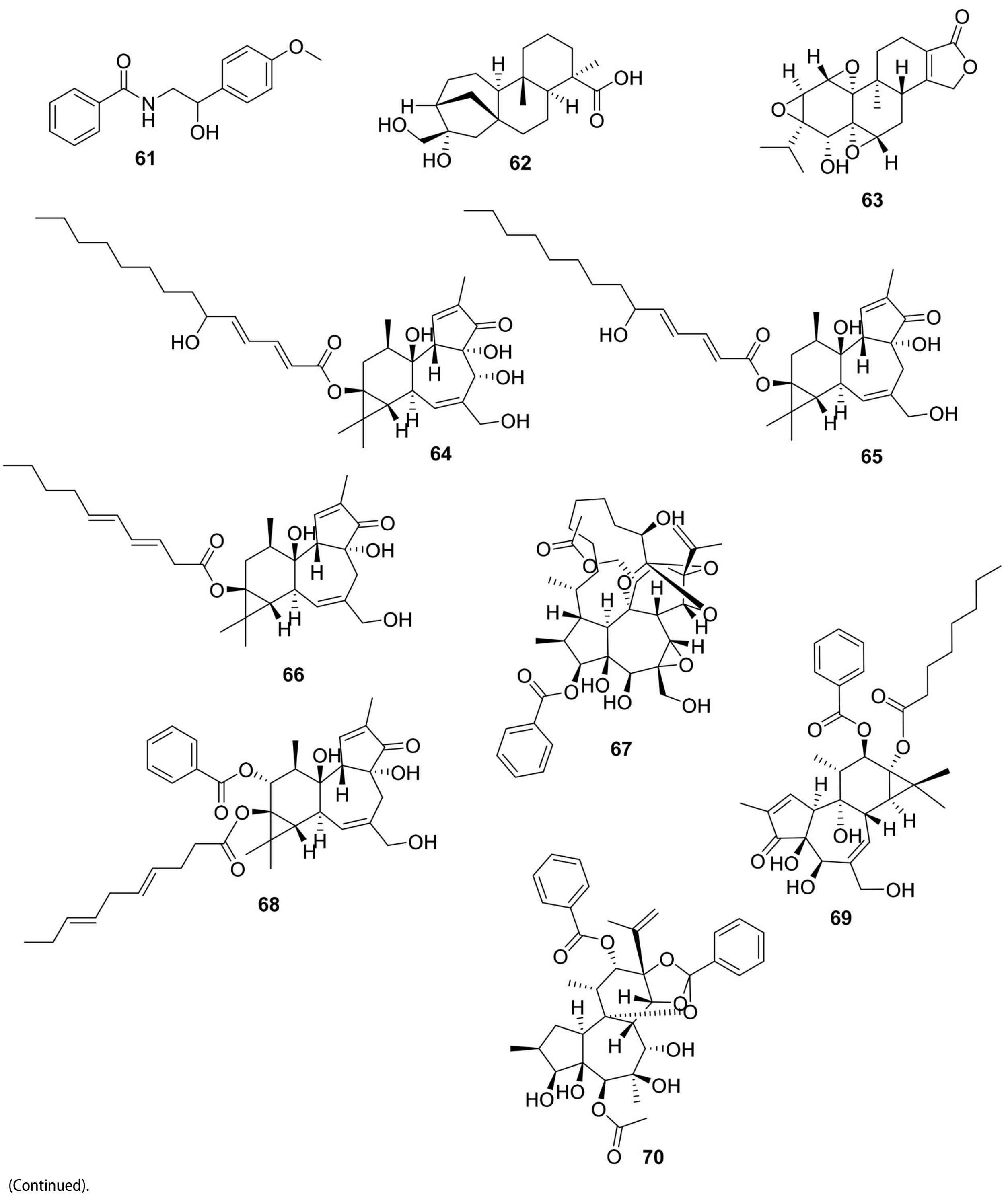

Figure 1. (Continued).

and loliolide interfere with virus entry in host cell (Hsu et al. 2015; Chung et al. 2016).

### *Inhibitors of virion attachment to host cells*

SARS-CoV-2 cell receptor angiotensin-converting enzyme mediates the fusion of viral and host membranes and this interaction is inhibited by oleanane triterpenes, oleanane saponins (Li et al. 2021), iridoid glycosides, such as loganic acid (Zhang et al. 2009), and phenolics such as such as tetra-*O*-galloyl-β-D-glucose, luteolin, oroxylin (Yi et al. 2004), epigallocatechin-3-gallate (Weber et al. 2015), procyanidin B2-di-gallate (Derksen et al. 2014), honokiol (Fang et al. 2015), emodin (**7**), rhein (Ho et al. 2007), 4,5-di-*O*-caffeoylquinic acid (**40**), oleandrin (**74**) (Mahmood et al. 1993; Singh et al. 2013) and ocotillone, the latter hampering the binding of HIV-1 envelope glycoprotein 20 to CD4 of T lymphocytes (Chang et al. 2003). Glycyrrhizin restrains HIV entry in the host cell by decreasing the fluidisation of the plasma membrane (Harada 2005). Panduratin A (**22**) acts at the pre-entry phase against SARS-CoV-2 (Kanjanasirirat et al. 2020) as well as the iridoid glycoside swertiamarin (Li et al. 2016).

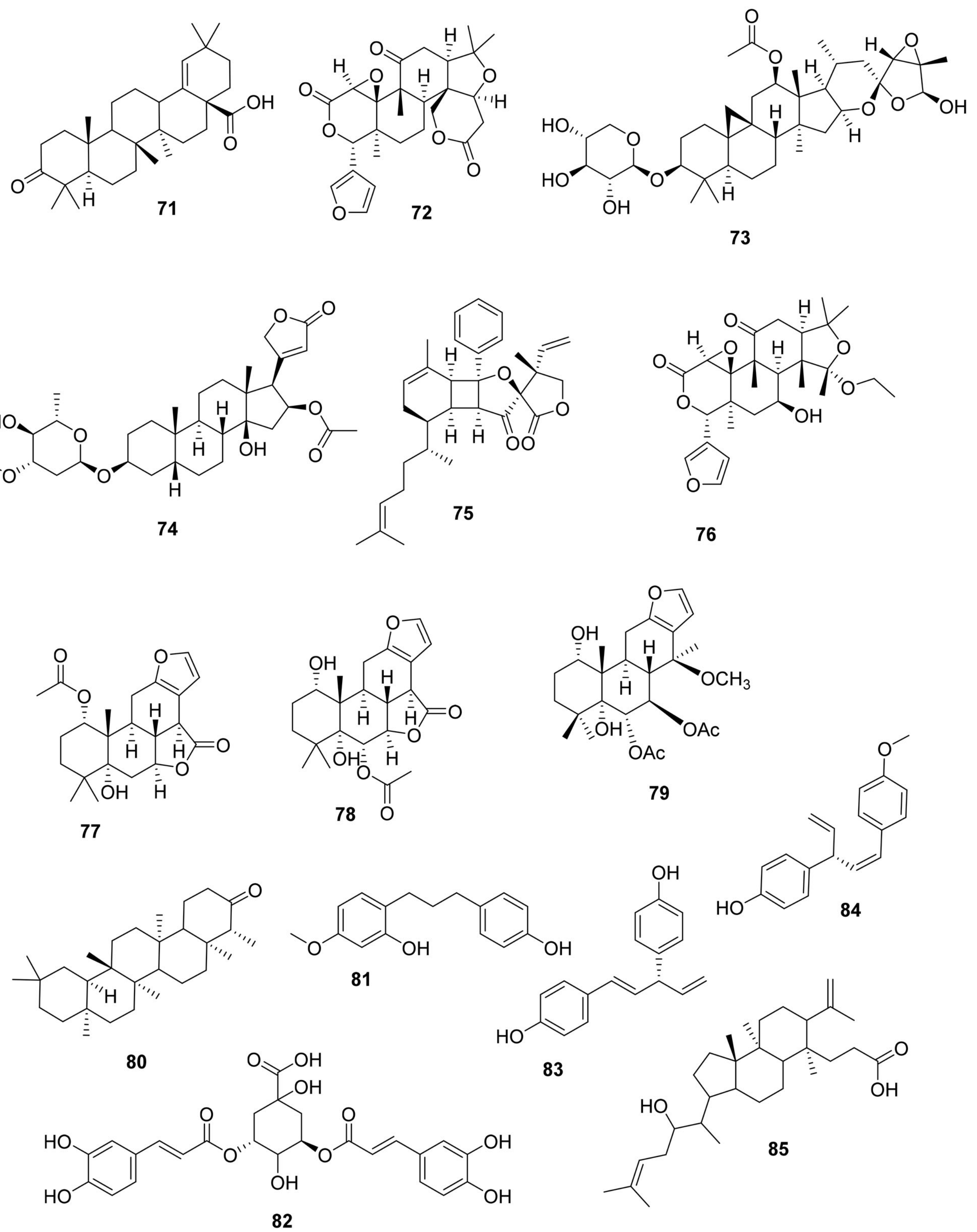

Figure 1. (Continued).

Triterpene saponins block the interaction between spike proteins and the angiotensin-converting enzyme. Of note, withanone very strongly inhibited the binding of spike proteins with angiotensin-converting enzyme and SARS-CoV ($IC_{50}$ 0.3 ng/mL) (Balkrishna et al. 2021). Interference with calcium host cell homeostasis also disturbs the expression of surface receptors to viruses (Huang et al. 2021). Alkaloids do not seem to act as virus attachment inhibitors.

### *Inhibitors of virus fusion and entry*

Natural products inhibiting virus attachment to host cells repress virus internalisation, such as tetra-*O*-galloyl-β-D-glucose (Yi et al. 2004). Agents blocking viral fusion are phenolics such as (+)-catechin gallate (Chang et al. 2016) and epigallocatechin-3-*O*-gallate (Derksen et al. 2014), or caffeic (Weng et al. 2019), tangeretin (Xu et al. 2014), or ellagic acid (Cui et al. 2018).

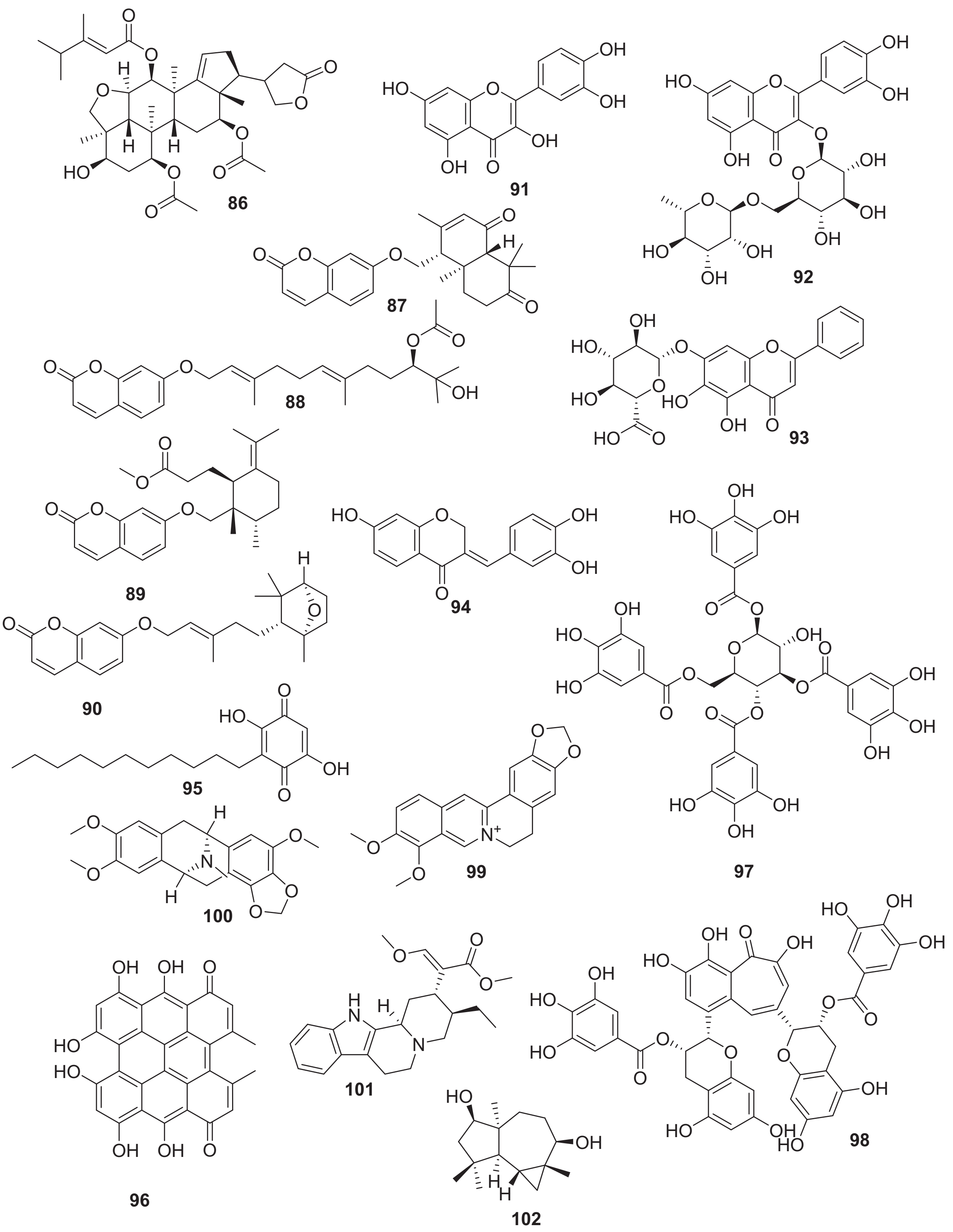

**Figure 1.** (Continued).

Trachelogenin (**31**) inhibited HCV entry in Huh7 cells by blocking glycoprotein E2 binding to CD81 (Qian et al. 2016). Capsaicin blocked LASV entry in host cells (Tang, Song, et al. 2020; Tang, Zhang, et al. 2020). Regarding saponins, they tend to block the entry of HIV in host cells as seen with arganine (Gosse et al. 2002) and cimicifugin with RSV (Xu et al. 2014). The precise mode of antiviral action of saponin is unknown but they may alter the outer envelope of enveloped viruses and compromise the anchoring of virus to host cells. Further, saponins bind to membranes cholesterol. The viral envelope contains cholesterol which promotes the fusion of viruses with host cells, and cholesterol in the host cell membrane promotes the fusion of the virion to the host cell (Ahn et al. 2002). Since saponins bind to membrane cholesterol it is possible to propose an anti-RNA virus mode of action of triterpene saponins and saponins at large which could involve, at least in part, binding to viral and host membrane cholesterol. In this light, it can be suggested that non-enveloped RNA viruses resist phenolics, saponins, and tannins because they are deprived of outer membranes. As for tannins, geraniin (**43**) suppressed HIV-1 entry in MT4 cells (Notka et al. 2003).

Cardiac glycosides such as ouabain and digitoxin are broad-spectrum antiviral molecules acting on the replication of the CHIKV, MERS and SARS-CoV via inhibition of $Na^+/K^+$-ATPase (Amarelle and Lecuona 2018; Yang et al. 2018). Inhibition of $Na^+/K^+$ ATPase by oleandrin (**74**) attenuates ZIKV infection in mice. Conessine inhibited ZIKV (strain PAN2016) ($IC_{50}$ 9.7 μM) (Lima et al. 2021) *via* ion channel inhibition. Kuranone inhibits virus-induced autophagic flux (Min et al. 2020). Ion channels are involved in the viral internalisation of several viruses including EBOV, and oleandrin (**74**), ouabain, digitoxin and other cardiac glycosides which block $Na^+/K^+$ ATPase suppress a broad spectrum of viruses (Amarelle and Lecuona 2018). Disturbance of host cell cytoplasmic membrane potential by blocking ion channels (Charlton et al. 2020) may account for the activities of alkaloids such as conessine (Lima et al. 2021; Shen et al. 2019). Conessine binds to G-protein coupled receptors and ligand-activated ion channels.

MERS-CoV translocates in the endolysosomal system of host cells *via* a mechanism involving $Ca^{2+}$-permeable channels. SARS-CoV encodes for 3a protein which forms ion channels that become incorporated into the membrane of the host cells. Flavonol glycosides inhibit 3a protein *in vitro*, such as tiliroside, kaempferol 3-*O*-arabinoside and afzelin. Berbamine is a calcium channel blocker that inhibits late-stage of Flavivirus replication (Chen, Yang, Huang, et al. 2020; Chen, Yang, Zhai, et al. 2020). Tetrandrine and fangchinoline (**21**) impair MERS-CoV translocation in the host cell and are known channel inhibitors (Charlton et al. 2020). Hence, natural products known for being calcium channel inhibitors have the potential to be anti-coronavirus agents. In coronaviruses, calcium-dependent nucleocapsid and non-structural protein 3 (nsp3) interactions are necessary for replication. Further, Fujioka et al. (2018) noted that IVA haemagglutinin binds a sialylated voltage-dependent calcium channel Cav1.2 to trigger increased cytoplasmic calcium and subsequent Influenza virus entry and replication. It can be inferred that disturbance of membrane potential can induce changes in cytoplasmic calcium homeostasis whence blockage of viral entry. Thus, calcium modulators, which are manifold among natural products from Asian medicinal plants need to be examined. One such modulator is palmatine which blocks ZIKV entry in Vero cells.

## Inhibitors of uncoating of the virus and release of the viral contents in the host cells

These are apparently few but ion channels are involved in pH modification of endosomes resulting in capsid disintegration in host cells suggesting that ion channels inhibitors have the potential to be antiviral.

## Translocation of naked virion to host cell nucleus

Podophyllotoxin, a well-known mitotic spindle poison of therapeutic value inhibits tubulin incorporation into microtubules. Consider that microtubules act as 'anchor' for viral capsid proteins to allow processes such as transport of naked virion to the nucleus, mRNA transcription, DNA replication and DNA packaging prior to virion release (Waye and Sing 2010) and it could be inferred that tubulin poisons have the potential to be developed into antiviral agents. Tubulin poisons among flowering plants in Asia and the Pacific include apocynaceous monoterpene alkaloids.

## Topoisomerase inhibitors

Planar heterocyclic indole alkaloids intercalate DNA and block topoisomerase. Topoisomerase is needed for (+)- RNA virus replication. The most active antiviral alkaloids are planar and as such may interact with viral DNA or DNA-processing enzymes such as topoisomerase (Xu et al. 2014). Manassantin B inhibits topoisomerase (Lee et al. 2009) and this mechanism could explain why it inhibits the replication of CV and HIV. Camptothecin stabilises topoisomerase I-DNA complex and inhibits the religation of DNA and therefore blocks virus replication. Protoberberines are DNA-stabilizing agent and this may explain their antiviral effects. In this light, carbazole alkaloids could be screened for their antiviral activities.

## Protease inhibitors

The genome of single-stranded (+)-RNA viruses is translated directly by host cell ribosomes into a polyprotein cleaved further by viral proteases into functional proteins. Protease inhibitors are mainly amphiphilic phenolics with low to medium molecular masses. For instance, flavonoids block MERS-CoV polyprotein cleavage such as 3′-(3-methylbut-2-enyl)-3′,4,7-trihydroxyflavane, papyriflavonol A (Park et al. 2017), herbacetin, quercetin 3-*O*-β-D-glucoside and isobavachalcone. Hydrolysable tannins such as 1,2,3,4,6-penta-*O*-galloyl-β-D-glucoside, 1,3,4,6-tetra-*O*-galloyl-β-D-glucoside (Zuo et al. 2005), 1, 2, 3, 6-tetragalloy1-β-D-glucopyranose, pentagalloyl glucose (Ajala et al. 2014), tellimagrandin I and II (Yue et al. 2006), casuarinin, corilagin and chebulagic acid are HCV proteases repressors (Ajala et al. 2014). Gallotannins, such as penta-*O*-galloyl-glucose and tetra-*O*-galloyl-glucose strongly inhibited CV protease (Abdel-Mageed et al. 2014). 1,3,5-trihydroxyxanthone, anolignan A (Pailee et al. 2018; Lee et al. 2010) and camelliatannin H ($IC_{50}$ 0.9 μM) (Park et al. 2002) block HIV protease. 5-Hydroxymethyl-(2,2′:5′,2″)-terthienyl tiglate and 21β-*O*-[(2E)-6-hydroxyl-2,6-dimethyl-2,7- octadienoyl] pitheduloside G (Nguyen et al. 2018) are HIV protease repressors.

In the case of coronaviruses, single-stranded (+)-RNA in host cell cytoplasm is translated into a polyprotein by host cell ribosomes that is cleaved by two viral proteases: the papain-like protease ($PL^{pro)}$ and the 3-chyomotrypsin-likeprotease ($3CL^{pro}$) into non-structural proteins. Cryptotanshinone ($IC_{50}$ 0.8. μM) (Park et al. 2012), tryptanthrin (**17**) (Tsai et al. 2020), and iguesterin (Ryu et al. 2010) are strong $PL^{pro}$ inhibitors. Other inhibitors are prenylated phenolics such as 3′-(3-methylbut-2-enyl)-3′,4,7-trihydroxyflavane, kazinol F (Park et al. 2017), the 3-acyl coumarin psoralidin (Kim et al. 2014), and with milder potencies neobavaisoflavone (Kim et al. 2014), savinin (Wen et al. 2007), papyriflavonol A (Park et al. 2017), epicatechin (**35**) (Chen et al. 2005), pectolinarin (Jo et al. 2020) and β-sitosterol (Lin et al. 2005).

In general, phenolics inhibit $3CL^{pro}$ such as baicalein ($IC_{50}$ 0.3 mM), bavachinin (Kim et al. 2014), luteolin, hesperetin (Lin et al. 2005), herbacetin (Jo et al. 2020), tomentin E (Cho et al. 2013), amentoflavone (Ryu et al. 2010), isobavachalcone (Kim et al. 2014), theaflavin-3,3′-digallate (**97**) (Chen et al. 2005) and epigallocatechin gallate. Other examples are caffeine, theophylline (Chen et al. 2005) and sinigrin (Lin et al. 2005). In the Flaviviridae, the protease NS3 ($NS3^{pro}$) catalyses the processing of polyproteins into individual functional proteins. Glycosylation of flavonols favours activity towards $NS3^{pro}$ as seen in hesperidin compared with its aglycone hesperetin (Eberle et al. 2021). Protoberberine alkaloids inhibit WNV $NS2B/NS3^{pro}$ such palmatine (Jia et al. 2010). ZIKV $NS2B/NS3^{pro}$ is blocked by hesperetin, myricetin, luteolin and pedalitin (Cataneo et al. 2019; Lima et al. 2021). Rutin (Zuo et al. 2005), kaempferol-3,7-bisrhamnoside (Yang et al. 2017), quercetin (**91**) (Zuo et al. 2005), ethylgallate, bergenin, gallic acid (**1**) and ellagic acid (Zuo et al. 2005; Modi et al. 2013). Honey and royal jelly natural products displayed some level of inhibition against SARS-CoV main protease (Zarei et al. 2021; Amirkhani et al. 2022).

## Polymerase inhibitors

RNA-dependent RNA polymerase catalyses the synthesis of a double-stranded RNA replicated into multiple (+)-RNA. This enzyme is a therapeutic target of drugs such as Remdesivir and is inhibited by phenolics, such as robinetin (Ahmed-Belkacem et al. 2014), myricetin (Ono et al. 1990), and usnic acid (Peyrat et al. 2016). Sennosides A and B strongly inhibited the HIV DNA polymerase (Esposito et al. 2016) as well as Amaryllidaceae alkaloids. Examples of terpenes inhibiting HIV DNA polymerase are pomolic acid, koetjapic acid (Sun et al. 1999), and gossypol. Platycodin D (**33**), platycodin D2 and platycodin D3 inhibited HCV NS5B polymerase (Kim et al. 2013). Natural products inhibiting RNA replication have mainly medium molecular masses and are principally amphiphilic.

For single-stranded (+)-RNA viruses, these inhibitors are chrysosplenetin (**14**), penduletin (**15**) (Zhu et al. 2011), catechin, pinocembrin (Le Lee et al. 2019), resveratrol (Lin et al. 2017), arborinine, pseudane IX, chalepin (Wahyun et al. 2014), lycorine (**3**) (Zou et al. 2009), tryptanthrin (**17**) (Tsai et al. 2020), cornuside (Zhang et al. 2021), celastrol (Yu et al. 2017), tatanan A (Yao et al. 2018), phenolic glycosides (Bourjot et al. 2012), trachelogenin (**31**), 3-hydroxy caruilignan C (Wu, Cheng, et al. 2012; Wu, Lin, et al. 2012) and 4,4′,7,7′-tetrahydroxy-2,2′, 8,8′-tetramethoxy-1,1′-di-phenanthrene with IVA. For single-stranded (-)-RNA viruses an example of polymerase inhibitor is tangeretin (Xu et al. 2014).

## Reverse transcriptase inhibitors

If the single-stranded (+)-RNA viruses are equipped with the reverse transcriptase enzyme, a complementary single-stranded DNA is produced which is made into double-stranded DNA integrating into the host DNA permanently. Inhibitors of reverse transcriptase *in vitro* are mainly low to medium molecular mass and hydrophilic or mid-polar phenolics such as gallic acid (**1**) (Modi et al. 2013), garciosine B (Pailee et al. 2018), pellucidin A (Thongphichai et al. 2019), 1,3,5-trihydroxyxanthone (Pailee et al. 2018), anolignan A (Rimando et al. 1994), manassantin A (Lee et al. 2010), 4,5-di-*O*-caffeoylquinic acid (**40**) (Mahmood et al. 1993), myricetin (Ortega et al. 2017), quercetin 3-*O*-(6″-feruloyl)-β-galactopyranoside (Datta et al. 2004), kaempferol-7-*O*-glucoside, dihydrokaempferol-3-*O*-rhamnoside (Wang et al. 2014), hinokiflavone, agathisflavone (Lin et al. 1997) and dihydroisomorellin (Reutrakul et al. 2006). With higher molecular masses are calanolide A (**41**) (Galinis et al. 1996), as well as tetragalloyl quinic (Nishizawa et al. 1989), 1,2,6-trigalloylglucopyranose, 1,2,3,6-tetragalloyl glucopyranose (Min et al. 2004) and geraniin (**43**) (Notka et al. 2003). Examples of terpenes inhibiting reverse transcriptase are viscoazulone (Datta et al. 2004), 16β-17-dihydroxy-*ent*-kauran-19-oic acid (**62**), limonin (**72**), nomilin, (24*E*)-3-oxo-lanostane-8,24-dien-26-oic acid (Zhang et al. 2003), betulinic acid (Reutrakul et al. 2010), garciosaterpene A, oleanolic acid, and andrographolide (Gurrapu and Mamidala 2017).

## Integrase inhibitors

HIV integrase inhibitors are phenolics such as apigenin 7-*O*-β-D-(4″-caffeoyl) glucuronide (Lee et al. 2003), kuwanon L (Esposito et al. 2015), orobol, lithospermic acid (Bailly and Cotelle 2005), catechin (Panthong et al. 2015), wedelolactone ($IC_{50}$ 4 μM), 1,3,6-tri-*O*-galloyl-β-D-glucopyranose, 1,2,3,4,6-penta-*O*-galloyl-β-D-glucopyranose (Ahn et al. 2002), chebulagic acid (Ajala et al. 2014) as proanthocyanidin A2 (Suedee et al. 2013).

## Ribonuclease inhibitors

These are anthraquinones such as rhein, sennosides A and B (Esposito et al. 2016) and naphthoquinones such as juglone (Min et al. 2002).

## Helicase inhibitors

In SARS-CoV, the viral helicase with nsp13 ATPase catalyses the generation of (-) strand RNA from (+)-RNA. Flavones such as myricetin and scutellarein ($IC_{50}$ 0.8 μM) are strong helicase inhibitors as well as ε-viniferin with HCV NS3 helicase ($IC_{50}$ 58.7 nM) (Lee et al. 2019).

## Protein synthesis inhibitors

Examples of protein synthesis inhibitors are wedelolactone, arborinine, pseudane IX (Manvar et al. 2012; Wahyun et al. 2014), lycorine (**3**), proanthocyanidins (Maeda et al. 2011), pristimerin and Amaryllidaceae alkaloids.

## Protein kinase inhibitors

The transcription of viral genetic material by enveloped viruses including α-viruses is regulated by phosphorylation mediated by the virus-associated protein kinase. Berberine (**98**) inhibited of mitogen-activated protein kinase signalling in host cells infected by CHIKV (Varghese et al. 2016). Inhibition of protein kinase C increases host cell death infected by SINV *in vitro* and we argue that the antiviral activity of 12-*O*-tetradecanoylphorbol 13-acetate (**34**) and other tiglianes towards viruses is due, at least in part, to protein kinase C activation. The protein Gag mediates the assembly and release of virus from an infected host cell and this mediation requires its phosphorylation by host cell protein kinase C and this may explain, at least in part, why natural products inhibiting protein kinase C such as tigliane diterpenes are often antiretrovial (Huang et al. 2014).

### *Assembly of viral genome and macromolecules into progeny virions*

Berberine (**98**) repressed IV protein trafficking/maturation.

### *Release of viral progeny*

The release of newly formed HIV virus from host cell requires cleaving of Gag protein by a viral protease (Freed 2015) which is blocked by ellagic acid (Modi et al. 2013). In (-)-RNA viruses, neuraminidase catalyses the hydrolysis of the α-(2,3)- or α-(2,6)-glycosidic linkage between a terminal sialic acid residue and its adjacent carbohydrate moiety on the host receptor allowing the release from hots cells of the newly formed viral progeny, and Oseltamivir is an example of neuraminidase inhibitor used for the treatment of influenza (Moscona 2005).

Neuraminidase inhibitors are mainly flavonoids including tamarixetin 3-robinobioside (Nguyen et al. 2016), myricetin-3′,5′-dimethylether 3-*O*-β-D-galactopyranoside, and agathisflavone (de Freitas et al. 2020; (+)-catechin gallate (Ide et al. 2016), epigallocatechin-3-*O*-gallate (Derksen et al. 2014), theaflavin-3,3′-digallate (**97**) and hispidulin (Bang et al. 2016). Other neuraminidase inhibitors are hydroxycinnamic acid derivatives such as rosmarinic acid methyl ester I, chlorogenic acid , forsythoside A (Law et al. 2017) as well as sappanone A (**94**), ε-viniferin (Nguyen et al. 2011), isocorilagin (Chen, Yang, Huang, et al. 2020; Chen, Yang, Zhai, et al. 2020) and forsypensins A-E.

### *Miscellaneous targets*

Andrographolide repressed IV by targeting host cell NF-*k*B. Fangchinoline (**21**) inhibited HIV glycoprotein 160 proteolytic processing (Wan et al. 2012). Triptolide (**63**) blocks HIV gene transcription and replication (Wan and Chen 2014). Lycorine (**3**) and dendrobine inhibit IV by interfering with nucleoprotein (Li et al. 2017).

## Structure-activity: the importance of planarity

In general, the more planar is a secondary metabolite of medium molecular mass the more specific the antiviral activities due to, at least in part, their ability to intercalate into viral genetic material and subsequent inhibition of enzymes targeting DNA and/or RNA. Regarding phenolics, the following observations can be made: (i) the presence of a quinone moiety boosts the activity of phenolics such as aloe-emodin (**2**) (Lin et al. 2008), 2-(1- hydroxyethyl)naphtho[2,3-b]furan-4,9-dione (Takegami et al. 1998), shikonin (Lin et al. 2008), plumbagin (**32**) as well as naphthalene framework such as diphyllin (**30**) (Cui et al. 2014) or hypericin (**96**) (Yasuda et al. 2010); (ii) Glycosylation of flavonols is detrimental for activity as seen in pectolinarin (Simões et al. 2011) and quercetrin (Chiow et al. 2016). Glycosylation of stilbenes translates in weaker potencies against IV such as piceid (Nguyen et al. 2011); (iii) Oligomerization of stilbenes is not detrimental to antiviral activity (Yang et al. 2005) neither is the condensation of flavonols in bisflavonols (de Freitas et al. 2020); (iv) Methoxylation of flavonols is detrimental to activity (Chen et al. 2019); (v) Introduction of ketone group in position 3 of flavans increases anti-HIV activity as seen with kuwanon L. ($EC_{50}$ 1.9 µM) (Esposito et al. 2015). Methoxylation of isoorientin is detrimental to activity against RSV (Chen et al. 2019). Planar indole alkaloids are often strongly retroviral (**49**) (Hsieh et al. 2004) via topoisomerase inhibition (Xu et al. 2014). Strong $PL^{pro}$ inhibitors are mainly planar (Park et al. 2012). Opening of the methylenedioxy moiety of Amaryllidaceae alkaloids is detrimental to activity towards DV (Zou et al. 2009).

## Natural products with the largest selectivity index

The S.I. is a very important characteristic for antiviral activity and it may be more important than the $IC_{50}$. Natural products from Angiosperms are often cytotoxic and a compound demonstrating S.I. of 10 or higher it is considered as prospective (Smee et al. 2017). This is the case for aloe-emodin (**2**), silvestrol (**8**), plumbagin (**32**), kuwanon G (**16**), panduratine A (**8**), hydroxytyrosol (**36**), lycorine (**3**), tylophorine (**20**), tryptanthrin (**17**), coclaurine (**44**), nornucoferine, drymaritine (**49**), uroslic acid, excoecafolin B (**64**) and C (**65**), trigonothyrin F (**70**), oleandrin (**74**), and biyouyanagin A (**75**). Natural products such as indole alkaloids or xanthones tend to be cytotoxic and have therefore low S.I. (Hosseinzadeh et al. 2018).

## Natural products active *in vivo*

Natural products with very strong activity *in vitro* are seldom active *in vivo* orally because of first-pass metabolism. In the case of flavonoids and hydroxycinnamic acids and especially saponins, oral bioavailability is in general modest, however, oral activity was observed with chlorogenic acid, forsythoside A (Law et al. 2017), cinnamic acid (Hayashi et al. 2007), tangeretin (Xu et al. 2015), isoorientin, homonojirimycin (Zhang, Liu, et al. 2013; Zhang, Li, et al. 2013) and polyphylla saponin I (Pu et al. 2015). Berberine (**98**) has low oral bioavailability and afforded levels of protection in mice infected with IVA (Shao et al. 2020). An example of sesquiterpene active *in vivo* orally is atractylon (Chen et al. 2016). Other instances are calycosin-7-*O*-β-D-glucopyranoside (Zhu et al. 2009), manassantin B (Song et al. 2019) and gymnemic acid A (Sinsheimer et al. 1968).

Compounds administered by injection do not undergo first-pass metabolism. Examples of phenolics and phenolic glycosides given intraperitoneally with antiviral activity are cirsitakaoside, dihydroquercetin (Galochkina et al. 2016), and ellagic acid (Park et al. 2013). Iridoid glycosides are often active intraperitoneally such as morroniside (Li et al. 2019) or geniposide (Zhang, Ai, et al. 2017; Zhang, Rumschlag-Booms, et al. 2017). Berbamine was effective intraperitoneally with JEV (Huang et al. 2021). Using the subcutaneous route, hippeastrine yielded some protection against ZIKV (Zhou et al. 2017) whereas pancratistatin (**25**) protected mice against JEV (Gabrielsen et al. 1992; Table 1).

## Concluding remarks

Compared with currently available antiviral drugs, natural products from Angiosperms in Asia and the Pacific appear to have numerous targets acting synergistically and at the various stages of RNA virus replication. The mechanisms of action of these principles *in vitro* are also dependent on their concentrations. Weinstein and Albersheim (1983) presented evidence that natural products from Angiosperms act non-specifically in order to avoid the development of resistance by phytopathogenic microbes (Amoros et al. 1992). This is one of the reasons why isolating an antiviral natural product from Angiosperms for clinical systemic use with a low therapeutic index and working at micromolar plasmatic concentration is an Augean task. Some natural products have reached clinical trials for HIV such as calanolide A (**41**) and others, like shikimic acid, are used as the starting material for the synthesis of antiviral drugs (Ramazani et al. 2021). It is, therefore, reasonable to anticipate the identification of a lead for the prevention and/or treatment of RNA virus infection from the medicinal Angiosperms of Asia and the Pacific. Hydroxytyrosol (**36**), silvestrol (**8**), lycorine (**3**), tylophorine (**20**) and 12-*O*-tetradecanoylphorbol 13-acetate (**34**) with $IC_{50}$ values below 0.01 µg/mL and S.I. above 100 (or their hemisynthetic derivatives) may have the potential to be developed as anti-RNA virus leads. In the rural settings of Southeast Asia and the Pacific, medicinal plants are used as herbal remedies for the treatment of COVID-19 and other viral diseases and some of them with proven efficacy, such as *A. paniculata* (Wanaratna et al. 2021) or *B. rotunda*. Another alternative would the development of extracts of fractions undergoing clinical trials and manufactured under strict pharmaceutical control. In this light, it is of the uttermost importance to preserve the primary rainforests of Asia and the Pacific and the medicinal traditional knowledge of indigenous tribes.

## Disclosure statement

No potential conflict of interest was reported by the authors.

## Funding

The author(s) reported there is no funding associated with the work featured in this article.
